# A Mitochondria‐Targeted Nanozyme Platform for Multi‐Pathway Tumor Therapy via Ferroptosis and Cuproptosis Regulation

**DOI:** 10.1002/advs.202417616

**Published:** 2025-06-25

**Authors:** Chenguang Liu, Lingxiao Guo, Yuying Cheng, Jingjie Gao, Hanling Pan, Jiayi Zhu, Danting Li, Liqing Jiao, Caiyun Fu

**Affiliations:** ^1^ College of Life Sciences and Medicine Zhejiang Sci‐Tech University Hangzhou 310018 P. R. China; ^2^ Zhejiang Provincial Engineering Research Center of New Technologies and Applications for Targeted Therapy of Major Diseases Zhejiang Sci‐Tech University Hangzhou 310018 P. R. China

**Keywords:** cuproptosis, ferroptosis, intracellular hemostasis, metal‐based nanocomposites, nanozyme

## Abstract

Transition metal‐based nanotherapeutics, such as chemodynamic therapy and ferroptosis‐ or cuproptosis‐induced strategies, hold great potential for cancer treatment. Copper‐ and iron‐based nanozymes enhance reactive oxygen species (ROS) generation and regulate metal ion homeostasis, driving ferroptosis and cuproptosis. However, simultaneous delivery of copper and iron ions and the role of mitochondria‐targeted copper in inducing cuproptosis remain underexplored. Here, a dual‐functional nano‐heterojunction platform, MIL‐Cu_1.8_S‐TPP/FA, is reproted, integrating iron‐ and copper‐based components for synergistic ferroptosis and cuproptosis induction. Mitochondria‐targeted Cu_1.8_S nanodots demonstrated high biocompatibility and efficiently induced cuproptosis by disrupting mitochondrial iron‐sulfur proteins. Combined with MIL‐88B, the iron‐based metal‐organic framework, the MIL‐Cu_1.8_S heterojunction exhibited enhanced ROS catalytic activity, confirmed by density functional theory (DFT) analysis, with improved H_2_O_2_ adsorption and lower energy barriers for peroxidase (POD)‐like reactions. The dual‐targeting MIL‐Cu_1.8_S‐TPP/FA nanoplatform effectively delivered copper ions to mitochondria and iron ions to tumor cells, modulating key ferroptosis‐ and cuproptosis‐related markers, such as GPX4, GSH, FDX‐1, and HSP70. The platform synergistically combined photothermal effects with multi‐pathway cell death mechanisms, achieving significant anti‐tumor efficacy in vitro and in vivo. This study underscores the therapeutic potential of synchronously delivering copper and iron ions and highlights mitochondria‐targeted strategies in advancing multi‐modal cancer therapies.

## Graphical abstract

This work reports a mitochondria‐targeted nanozyme platform, MIL‐Cu_1.8_S‐TPP/FA, which induces potent cuproptosis via localized delivery of Cu_1.8_S nanodots to mitochondria. Surface‐anchoring these nanodots onto MIL‐88B metal‐organic frameworks enhances both passive and active tumor targeting. The resulting heterojunction structure exhibits synergistic POD‐like and GPx‐like catalytic activity, enabling coordinated disruption of iron and mitochondrial copper homeostasis to trigger ferroptosis and cuproptosis in tumor cells.

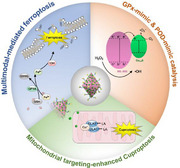

## 1 Introduction

Ion‐interference therapy (IIT) is an emerging therapeutic strategy that aims to regulate ion homeostasis within target cells or specific microenvironments to induce desired biological effects.^[^ ^1^ ^]^ In the context of cancer, the targeted disruption of metal ion homeostasis offers a promising approach to trigger regulated cell death pathways and circumvent the limitations of traditional chemotherapy, including multidrug resistance.^[^ ^2^ ^]^ Among various bioactive ions, iron and copper are particularly notable due to their distinct involvement in two mechanistically different but therapeutically relevant cell death pathways: ferroptosis and cuproptosis.^[^ ^3^ ^]^

Ferroptosis is defined as an iron‐dependent non‐apoptotic cell death resulting from extensive lipid peroxidation (LPO).^[^ ^3b^ ^]^ Particularly, Fe^2+^ ions catalyzed the Fenton reaction to generate hydroxyl radical (•OH), which further induces lipid peroxides (PLOOH) accumulation in the cell membrane along with Fe^2+^ ions. Glutathione peroxidase 4 (GPX4) can detoxify PLOOH into corresponding alcohol (PLOH) to protect cells from ferroptosis.^[^ ^4^ ^]^ Whereas, glutathione (GSH) can be oxidated into glutathione oxidized (GSSG) under the catalyzation of Fe^3+^ ions, which induces dysfunction of GPX4 in PLOOH detoxification. Similarly, the •OH catalysis and GPX4‐GSH ferroptosis defense system can be mediated via non‐ferrous ions metal ions such as Mn^x+^/Mn^x+1^, Co^+^/Co^3+^, Cu^+^/Cu^2+^, and Ce^3+^/Ce^4+^.^[^ ^5^ ^]^ Except GPX4‐GSH system, however, cellular ferroptosis can also be relieved FSP1‐CoQH2 system,^[^ ^6^ ^]^ suggesting that cancer cells are still resistant to ferroptosis even though introduce extra intracellular related metal ions. In contrast, cuproptosis is a novel mode of cell death caused by copper ions leading to a loss of iron‐sulfur cluster (Fe‐S) proteins and aggregation of dihydrolipoamide S‐acetyltransferase (DLAT).^[^ ^3a^ ^]^ Leveraging copper ions as an anti‐tumor strategy holds significant promise, as they not only modulate the GPX4‐GSH axis but also initiate cellular cuproptosis. This dual functionality synergistically activates downstream Fe^2+^‐dependent LPO cascades, i.e., PLOOH‐PLOO• reaction. Such a multi‐pathway, cross‐activation approach provides a compelling and innovative framework for advancing cancer therapeutics. Recent studies have begun to explore copper‐ and iron‐based nanomaterials for combinatorial induction of ferroptosis and cuproptosis.^[^ ^7^ ^]^ However, very few have investigated whether controlling the intracellular fate and sub‐organelle targeting of metal ions could further improve therapeutic precision and efficacy.

**Figure 1:**
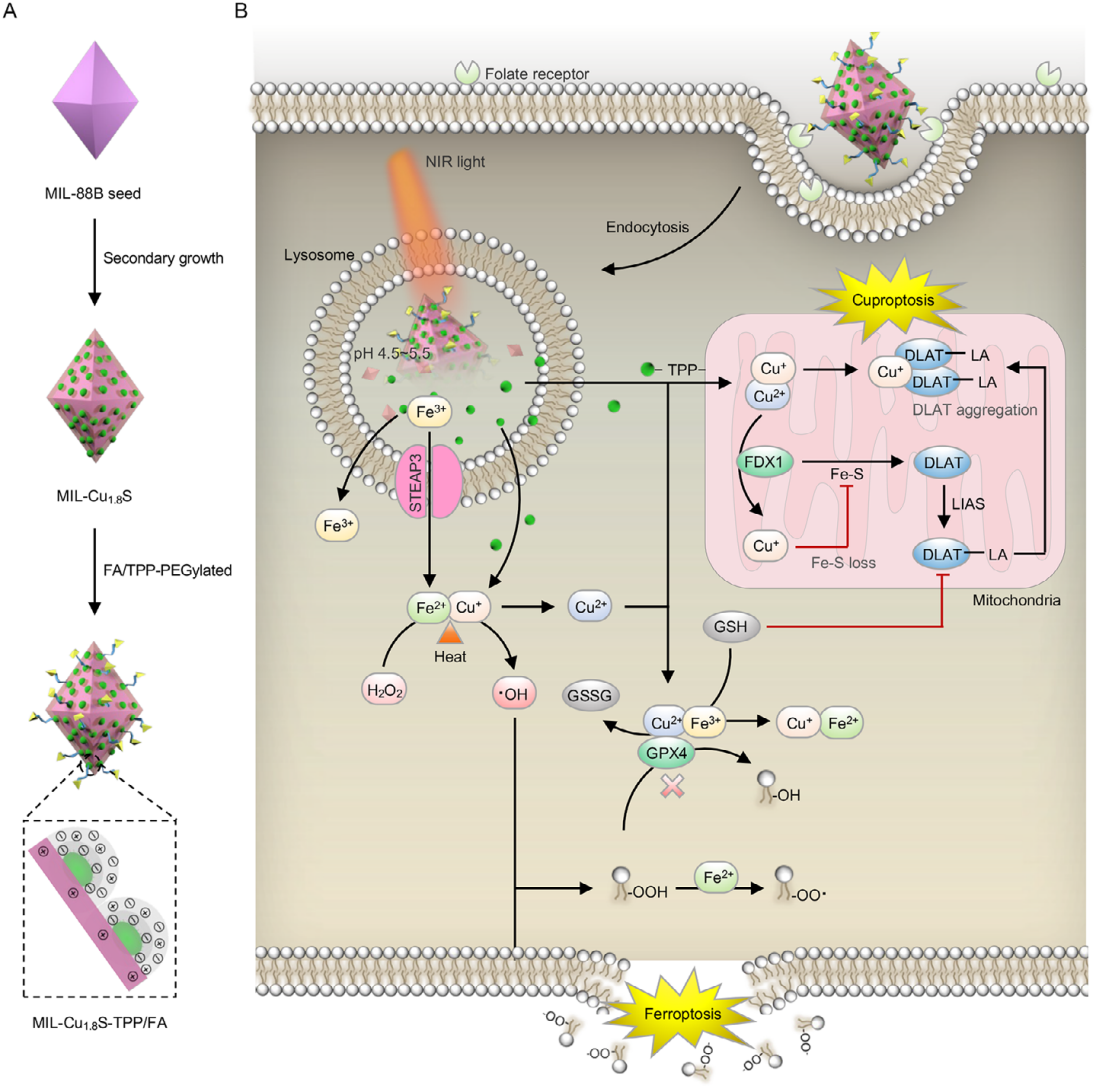
Illustration of A) preparation of MIL‐Cu_1.8_S‐TPP/FA and B) their anti‐cancer processes in cancer cells under an irradiation of NIR laser, including pH‐responsive and photothermal‐enhanced metal ions release, further resulting in ferroptosis and cuproptosis cross‐regulations by iron and copper ions.

Inspiring by the close correlation between cuproptosis and mitochondria, including the mitochondrial tricarboxylic acid (TCA) cycle, respiratory chain, and Fe‐S proteins, we initially investigated the effect of mitochondria‐targeted delivery of copper ions on cuproptosis by using copper sulfide (Cu_1.8_S) nanodots, which contains copper ions in two valence states. To further improve the biodistribution and tumor enrichment caused by the nanoscale effect and surface physicochemical properties, and also to explore the effects of copper and iron bimetallic homeostatic regulation on cell death, we constructed an engineered nanocomposite with an iron‐based metal‐organic framework (MOF) Materials of Institute Lavoisier‐88B (MIL‐88B) as the main structure and Cu_1.8_S nanodots as embellishments (defined as MIL‐Cu_1.8_S) via a secondary‐growth method (**Figure** **1**A). Subsequently, as‐obtained nanocomposites were surface‐modified by folic acid (FA) and triphenylphosphine (TPP) for secondary targeting to cancer cells and mitochondria cancer and mitochondria target respectively (defined as MIL‐Cu_1.8_S‐TPP/FA). As illustrated in Figure 1B, targeted delivery of MIL‐Cu_1.8_S‐TPP/FA into cancer cells resulted in MIL‐88B cleavage in response to both exogenous near‐infrared (NIR) light and lysosomal low pH, releasing Fe^3+^ ions and Cu_1.8_S nanodots. Then these released components rapidly generated reactive oxygen species (ROS) in targeting cells, modulating the GPX4‐GSH axis, affecting mitochondrial function, and ultimately inducing ferroptosis and cuproptosis. MIL‐Cu_1.8_S‐TPP/FA was also investigated for its in vivo distribution and anti‐tumor effects. Together, MIL‐Cu_1.8_S‐TPP/FA constructed by a secondary‐growth method exhibited good tumor enrichment, photothermal‐enhanced intracellular ROS generation, and anti‐tumor effect via cross‐mediating ferroptosis and cuproptosis in vitro and in vivo.

## 2 Results and Discussion

### 2.1 Regulation of Cellular Cuproptosis by Cu_1.8_S Nanodot

**Figure 2:**
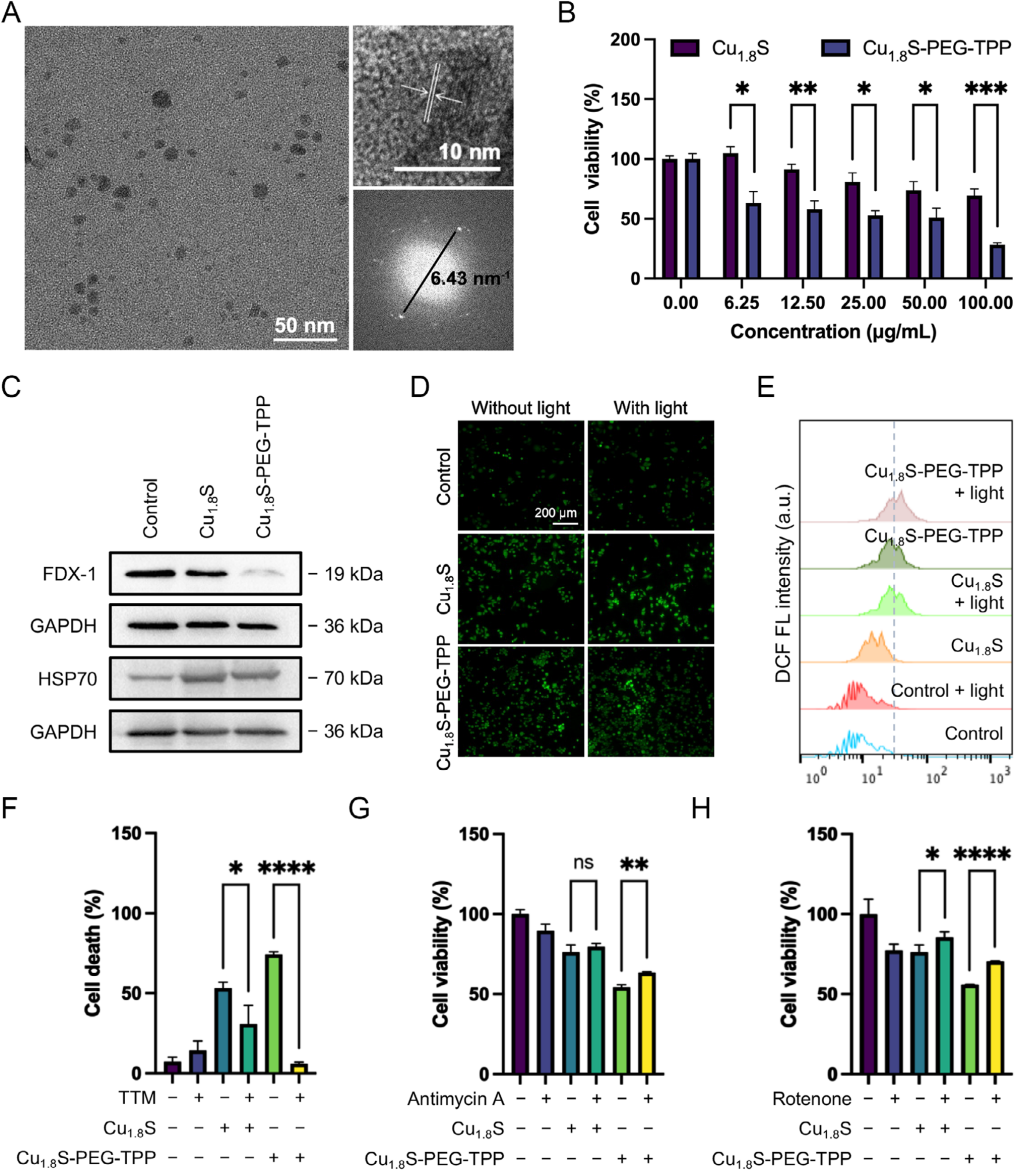
Mitochondria‐targeting modification endow Cu_1.8_S a cuproptosis‐inducing enhancement in 4T1 cells. A) TEM images of Cu_1.8_S nanodots. B) Cell viability of 4T1 cells treated with Cu_1.8_S or Cu_1.8_S‐PEG‐TPP. C) Western blot of cuproptosis‐related protein FDX‐1 and HSP70 treated with Cu_1.8_S and Cu_1.8_S‐PEG‐TPP. In vitro ROS generation was observed by D) CLSM images and E) flow cytometry. F) Cell death of 4T1 cells pretreated with copper chelation agent TTM and then treated with Cu_1.8_S or Cu_1.8_S‐PEG‐TPP. Cell viabilities of 4T1 cells pretreated with G) Antimycin A and H) Rotenone, and then treated with Cu_1.8_S or Cu_1.8_S‐PEG‐TPP. * *P* < 0.05, ** *P* < 0.01, *** *P* < 0.001, **** *P* < 0.0001.

It has been reported that Cu‐based nanomaterials, such as Cu nanocoordination,^[^ ^8^ ^]^ Cu^2+^ doped metal‐organic frameworks,^[^ ^9^ ^]^ Cu^2+^‐based nanocomplexes,^[^ ^10^ ^]^ etc., provided a novel strategy for cancer therapy by inducing cellular cuproptosis. Inspiring from the close relationship between cuproptosis and mitochondria, we endowed Cu_1.8_S nanodots, a typical copper‐based multi‐functional nanomaterials, with a mitochondria‐targeting capability by surface‐modifying thiol‐polyethylene glycol−TPP (HS−PEG−TPP). The Fourier transform infrared spectroscopy (FTIR) was employed to conform the modification of TPP on Cu_1.8_S nanodot. As shown in Figure S1 (Supporting Information), the peak at ≈1660 and 1296 cm^−1^ in the spectra of Cu_1.8_S nanodot can be ascribed to C═O stretching and C−N stretching of polyvinylpyrrolidone (PVP) that was used to assist in the synthesis of Cu_1.8_S nanodot.^[^ ^11^ ^]^ With PVP taking place by HS−PEG−NH_2_ due to the high affinity of copper and −SH, the peak at ≈1660 cm^−1^ decreases significantly. The peaks at ≈1635 and 997 cm^−1^ in the spectra of Cu_1.8_S‐PEG‐TPP correspond to C═O and C−P stretching in −O═C−TPP,^[^ ^12^ ^]^ indicating the linking of HOOC−TPP and Cu_1.8_S‐PEG‐NH_2_. The obtained Cu_1.8_S‐PEG‐TPP was observed by a transmission electron microscope (TEM). The Cu_1.8_S‐PEG‐TPP exhibit homogeneous and monodisperse particles with a size of ≈10 nm and maintain the lattice structure of Cu_1.8_S nanodots (**Figure** **2**A).

To investigate the effect of mitochondria‐targeting delivery of Cu_1.8_S nanodots on cellular cuproptosis, we first compared the cell inhabitation of Cu_1.8_S with/without mitochondria‐targeting modification by cell counting kit 8 (CCK‐8) assay. As shown in Figure 2B, the Cu_1.8_S shows a low cytotoxicity in mice breast cancer cells (4T1) at concentrations below 100 µg mL^−1^ as previously reported.^[^ ^13^ ^]^ In contrast, the cell viabilities of 4T1 cells after 24 h treatment of Cu_1.8_S with mitochondria‐targeting modification, i.e., Cu_1.8_S‐PEG‐TPP, are significantly lower, where the cell viability is 37.61% ± 1.74% at 100 µg mL^−1^. Notably, the TPP modification has no substantial influence on the photothermal property of Cu_1.8_S. Cu_1.8_S‐PEG‐TPP still exhibited a wide NIR absorption (700–1100 nm) and photothermal conversion under an 808‐nm laser irradiation (Figures S2 and S3, Supporting Information).

Furthermore, the possible anti‐cancer mechanism related to cuproptosis was explored. Western blot analysis revealed a notable decrease in the protein level of FDX‐1 and an increase in HSP70 expression in the Cu_1.8_S‐PEG‐TPP treatment group (Figure 2C; Figure S4, Supporting Information). FDX‐1 expression showed a slight decrease in cells treated with Cu_1.8_S compared to Cu_1.8_S‐PEG‐TPP, indicating that mitochondria‐targeting modification could promote the occurrence of cuproptosis. Meanwhile, the protein level of HSP70 in Cu_1.8_S and Cu_1.8_S‐PEG‐TPP increased, demonstrating cellular stress. Mass spectrometric analysis also revealed that Cu_1.8_S‐PEG‐TPP leads to the loss of Fe‐S cluster proteins (e.g., FDX‐1, LIAS, POLD1, and SDHB) in an FDX‐1‐dependent manner and HSP70 (Figure S5, Supporting Information). Confocal laser scanning microscope (CLSM) and flow cytometry results demonstrated that Cu_1.8_S‐PEG‐TPP more effectively enhanced intracellular ROS levels compared to Cu_1.8_S nanodots, and the ROS generation was further amplified under photothermal treatment (Figure 2D,E).

The cuproptosis induced by Cu_1.8_S‐PEG‐TPP in 4T1 cells was further determined using the copper chelator tetrathiomolybdate (TTM) and mitochondrial electron transport inhibitors antimycin A and rotenone. Antimycin A and rotenone, as inhibitors of mitochondrial complexes III and I, respectively, impair mitochondrial respiration and reduce electron flow through the respiratory chain, effectively lowering the demand for Fe─S proteins and reducing lipoylation activity.^[^ ^3^ ^]^ Treatment with cuproptosis or cuproptosis‐related inhibitors significantly rescued the Cu_1.8_S‐PEG‐TPP‐induced inhibition of cell viability (Figure 2F−H; Figures S6 and S7, Supporting Information), indicating that Cu_1.8_S‐PEG‐TPP suppresses 4T1 cells by modulating cuproptosis and impairing mitochondrial respiration. Similar results were also shown in the MB‐MDA‐231 cell line (Figure S8, Supporting Information).

### 2.2 Preparation and Characterization of MIL‐Cu_1.8_S‐TPP/FA

**Figure 3:**
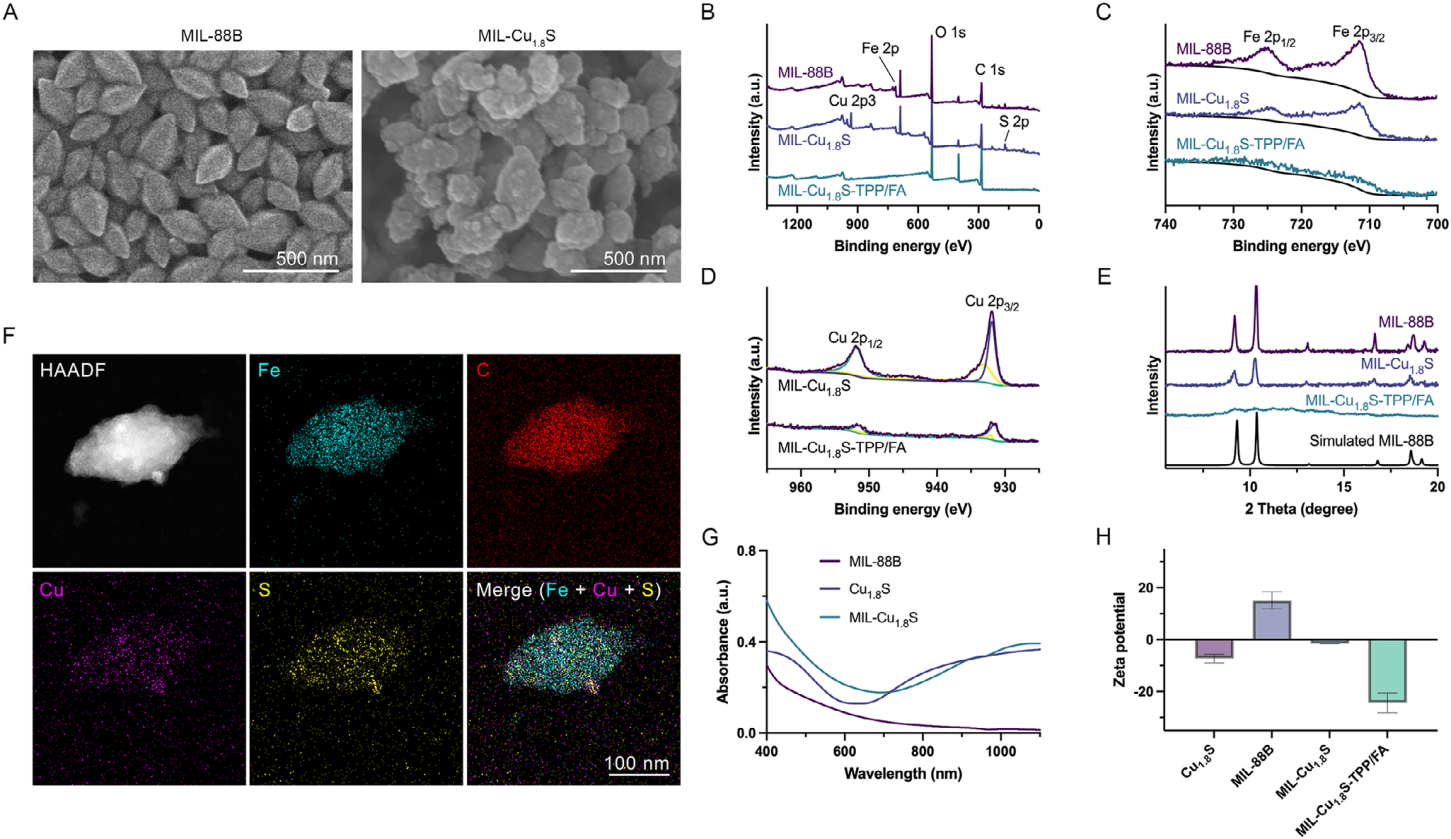
Preparation and characterization of MIL‐Cu_1.8_S‐TPP/FA. A) SEM images of MIL‐88B and MIL‐Cu_1.8_S. B) XPS spectrum of MIL‐88B, MIL‐Cu_1.8_S and MIL‐Cu_1.8_S‐TPP/FA C) XPS Fe 2p regions of MIL‐88B, MIL‐Cu_1.8_S and MIL‐Cu_1.8_S‐TPP/FA. D) XPS Cu 2p regions MIL‐Cu_1.8_S and MIL‐Cu_1.8_S‐TPP/FA. E) XRD patterns of MIL‐88B and MIL‐Cu_1.8_S and MIL‐Cu_1.8_S‐TPP/FA. F) EDS mapping images of MIL‐Cu_1.8_S‐TPP/FA. G) UV−vis absorption spectra of MIL‐88B, Cu_1.8_S, and MIL‐Cu_1.8_S. H) Zeta potential of Cu_1.8_S, MIL‐88B, MIL‐Cu_1.8_S and MIL‐Cu_1.8_S‐TPP/FA.

Considering to biodistribution and biosafety of nanomaterials according to the particle size and physicochemical properties,^[^ ^14^ ^]^ as well as the advantages of copper in ferroptosis‐like cell death, the Cu_1.8_S‐PEG‐TPP with cuproptosis‐regulating capability was combined to an acid‐sensitive MIL‐88B. To efficiently exploit the photo/chemical properties of Cu_1.8_S, maintain the ease of modification of the nanoparticles, and construct semiconductor‐metal heterojunctions, we employed the secondary growth method to distribute Cu_1.8_S nanodots on the surface of a MIL‐88B. As illustrated in Figure 1A, a MIL‐88B seed is synthesized, and then the obtained Cu_1.8_S nanodots were added in the secondary growth process, where the PVP and electrical double layer on the surface of Cu_1.8_S nanodots spatially limited their distribution on the MIL‐88B surface, thus avoiding aggregation.^[^ ^13^, ^15^ ^]^ By this secondary‐growth method, the MIL‐88B showed an octahedral spindle nanostructure (**Figure** **3**A). The thickness of the second‐grown MIL‐88B was controlled to a thin range by the concentration of FeCl_3_ and 2‐aminoterephthalic acid (H_2_N‐BDC) during growth, thus Cu_1.8_S nanodots remained exposed on the surface of the composite particles, facilitating subsequent FA/TPP‐PEGylated modification. The size of the resulting nanocomposites MIL‐Cu_1.8_S‐TPP/FA was 300.03 nm ± 0.25 nm (length) × 170.73 nm ± 25.26 nm (width). The composition details were also reflected from X‐ray photoelectron spectroscopy (XPS), showing that MIL‐Cu_1.8_S were composed of Cu, S, Fe, O, and C elements (Figure 3B). Iron and copper in MIL‐Cu_1.8_S remained intrinsically the same with free MIL‐88B and Cu_1.8_S nanodots as the valence state showed high similarity (Figure 3C,D). Meanwhile, XPS analysis of MIL‐Cu_1.8_S‐TPP/FA revealed significantly attenuated Fe 2p and Cu 2p signals compared to the unmodified MIL‐Cu_1.8_S, accompanied by enhanced C 1s and O 1s signals (Figure 3B–D). This change can be attributed to the surface passivation effect caused by the dense coverage of PEG‐TPP/FA ligands, which shield the underlying metal elements from detection due to the surface‐sensitive nature of XPS. Notably, such attenuation of metal signals is commonly observed in PEGylated and ligand‐functionalized nanomaterials and does not reflect elemental loss or structural degradation. The core composition and crystallinity were confirmed to remain intact by X‐ray diffraction (XRD) and energy dispersive spectrometer (EDS) mapping analysis. Based on XRD patterns (Figure 3E), both MIL‐88B and MIL‐Cu_1.8_S appeared similar packs to the stimulated MIL‐88B, indicating the structural intactness of MIL‐88B during the process though its crystallinity was reduced due to the introduction of Cu_1.8_S nanodots. Owning to the ultra‐small particle size of Cu_1.8_S nanodots and thus the low number of crystal stacking layers, the peaks attributed by Cu_1.8_S nanodots in MIL‐Cu_1.8_S sample widened and weakened, i.e., the broadening effect.^[^ ^16^ ^]^ The XRD patterns of MIL‐Cu_1.8_S‐TPP/FA displayed a phenomenon consistent with the XPS results, namely a significant reduction or near disappearance of characteristic diffraction peaks compared to unmodified MIL‐Cu_1.8_S. This decrease is primarily due to the surface conjugation of PEG‐FA and PEG‐TPP, which forms a disordered organic shell and disrupts the ordered packing of the crystal lattice, thereby weakening the long‐range crystallinity detectable by XRD. Despite this, EDS elemental mapping confirmed the structural integrity of MIL‐Cu_1.8_S‐TPP/FA. Specifically, Fe and C elements were uniformly distributed throughout the entire structure, while Cu and S elements appeared as discrete, co‐localized dots, with the overlap of purple and yellow signals forming white regions (Figure 3F). The ultraviolet and visible (UV−vis) spectra of MIL‐Cu_1.8_S show the characteristic absorption of broad absorption in the NIR region (Figure 3G). The Zeta potential value of MIL‐Cu_1.8_S was −1.56 mV ± 0.05 mV. After surface‐modified with FA and TPP (the modified nanocomposites were defined as MIL‐Cu_1.8_S ‐TPP/FA), the Zeta potential value of MIL‐Cu_1.8_S‐TPP/FA decreased to −24.37 mV ± 3.85 mV (Figure 3H). Inductively coupled plasma optical emission spectrometer (ICP‐OES) was used to determine the weights (%) of Fe and Cu in MIL‐Cu_1.8_S‐TPP/FA, where the proportion of Cu_1.8_S in the whole particle was calculated to 24.79% (w/w). In general, the MIL‐Cu_1.8_S‐TPP/FA was successfully synthesized with uniform morphology, good crystallinity, and well surface‐modified with Cu_1.8_S nanodots exposed on the surface of nanocomposites by a seed‐secondary‐growth method.

### 2.3 Effects and Mechanisms of Heterogeneous Junction‐Mediated ROS Generation

**Figure 4:**
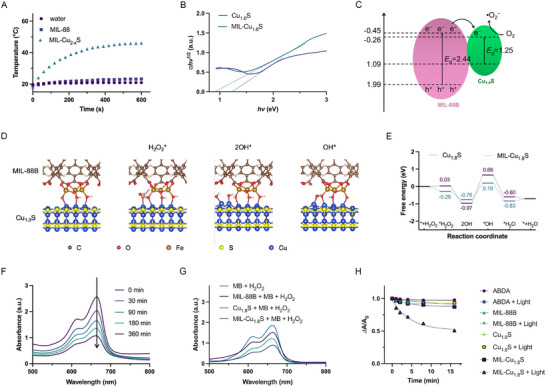
Enhanced ROS‐generating investigation of MIL‐Cu_1.8_S. A) Temperature curves of water, MIL‐88B, and MIL‐Cu_1.8_S aqueous dispersion exposed on 808 nm laser (1.5 W cm^−2^). B) The (Ahν)^1/2^ versus hν curve of Cu_1.8_S and MIL‐Cu_1.8_S. C) A plausible diagram for separation of electron‐hole pairs in the MIL‐Cu_1.8_S type‐I heterojunction. D) MIL‐Cu_1.8_S models in the prior H_2_O_2_‐dissociation process. E) Free energy diagrams of Cu_1.8_S and MIL‐Cu_1.8_S models in the POD‐mimic catalysis process. F) MB degradation after co‐culture with MIL‐88B, C_2‐x_S, and MIL‐Cu_1.8_S at 37 °C for 6 h, and G) Absorption spectrums of MB with MIL‐Cu_1.8_S co‐culture for different times. H) ^1^O_2_ generation was calculated from the absorbance of the ABDA residue at 378 nm under different conditions, respectively.

In the composite structure of MIL‐Cu_1.8_S, Cu_1.8_S nanodots partially combine with MIL‐88B and are inlaid on the surface of MIL‐88B. MIL‐Cu_1.8_S inherits the broad absorption and good properties of photothermal conversion of Cu_1.8_S nanodots. Under an irradiation of 808 nm laser (1.5 W cm^−2^), the MIL‐Cu_1.8_S aqueous suspension was heated to 46.0 °C, while there are few temperature changes in water and MIL‐88B aqueous suspension (**Figure** **4**A). To assess the energy conversion efficiency, the photothermal conversion efficiency (*η*) was calculated according to the total energy balance of the system by using the following equation:^[^ ^17^ ^]^

(1) $$\eta =\frac{\mathrm{hA}\left(\Delta T_{\max,\min}-\Delta T_{\max,H_{2}O}\right)}{I\left(1-10^{-A_{\lambda }}\right)}$$

where *I* refer to the laser power, *A_λ_* refers to the absorbance of samples at 808 nm, h refers to the heat transfer coefficient, *A* refers to the surface area of the container, and *ΔT* refers to the temperature change.

When Cu_1.8_S grew on the surface of MIL‐88 at a suitable density, the obtained MIL‐Cu_1.8_S showed a higher photothermal conversion efficiency (49.35%) than Cu_1.8_S nanodots (43.94%) at the same concentration (Figure S9, Supporting Information). With a higher concentration of Cu_1.8_S in the system (MIL‐C_1.8_S‐2 and MIL‐C_1.8_S‐3, Figure S9, Supporting Information), the photothermal conversion efficiency of obtained high Cu_1.8_S‐proportion MIL‐Cu_1.8_S decreased from 49.35% to 41.89%. Notably, the TPP/FA surface modification rarely affects the photothermal property of MIL‐Cu_1.8_S, in which the heating effect of MIL‐C_1.8_S‐TPP/FA was not significantly different from MIL‐Cu_1.8_S (Figure S10, Supporting Information).

More importantly, combining MIL‐88B and Cu_1.8_S enhances the photodynamic effect. The mechanism of ROS generation was deduced according to the energy band structure. As shown in Figure S11A,B (Supporting Information), the absorption spectrums of MIL‐88B and Cu_1.8_S were analyzed by the B. Tauc plot of the modified Kubelka‐Munk function plot method. The band gaps (E_g_) of MIL‐88B and Cu_1.8_S were surveyed by the curve of (αhν)^2^‐hν and (αhν)^1/2^‐hν,^[^ ^18^ ^]^ revealing 2.44 eV (MIL‐88B) and 1.25 eV (Cu_1.8_S). Then, the valence band positions of MIL‐88B and Cu_1.8_S were calculated based on the valence band‐XPS (Figure S11C,D, Supporting Information). Meanwhile, the band gap of MIL‐Cu_1.8_S was also calculated by the Kubelka‐Munk formula. The band gap of MIL‐Cu_1.8_S (0.92 eV) was narrower than that of the Cu_1.8_S (Figure 4B). It has been demonstrated that the narrow band gap can make the electron‐hole separation easier, thereby significantly improving the ROS generation during photodynamic therapy.^[^ ^19^ ^]^ As illustrated in Figure 4C, it is feasible to construct a p‐n junction with MIL‐88B, an n‐type semiconductor, and Cu_1.8_S, a p‐type semiconductor,^[^ ^20^ ^]^ by which the electronic structure through the synergistic effect can be effectively adjusted, promoting the separation efficiency of electron‐hole pairs under NIR irradiation and enhancing the phototherapy.

To elucidate the catalysis mechanism of MIL‐Cu_1.8_S and further provide clear insight into the correlation between their enzyme‐mimicking activities and our designed defective configuration, DFT calculations were employed and Figure 4D and Figure S12 (Supporting Information) show the critical intermediate structures along the reaction path during the H_2_O_2_ decomposition process in MIL‐Cu_1.8_S and Cu_1.8_S. The Cu and Fe in the interfaces of MIL‐Cu_1.8_S held strong coupling with the O‐bearing substrates and served as the preferred binding site for the homolysis of the H_2_O_2_* entity into 2OH* intermediate simultaneously, which activated this reaction thermodynamically. The H_2_O_2_ adsorption free energy (ΔG_H2O2*_) of Cu sites on the surface of MIL‐Cu_1.8_S heterojunction (‐0.29 eV) was more enhanced than that of Cu_1.8_S (0.03 eV), promoting the catalytic reaction due to the enrichment of H_2_O_2_. As shown in Figure 4E, the calculated energy diagrams indicated that the generation of free OH* species from 2OH* was the rate‐determining step of the whole •OH generation process. Because the adsorption strength of OH* on the surface slightly decreases to a moderate level, MIL‐Cu_1.8_S heterojunction had a low energy barrier for this reaction. Then, the generated OH* species produced •OH.

Combining these ROS generating mechanisms, the ROS generation through MIL‐Cu_1.8_S heterojunction is mainly contributed by several parts: 1) MIL‐Cu_1.8_S heterojunction exhibited peroxidase (POD)‐like enzymatic effect, promoting •OH generation. 2) The heterojunction construction facilitated the electron‐hole separation and transferring, during which singlet oxygen (^1^O_2_) is generated.^[^ ^21^ ^]^ 3) Fe^3+^ in MIL‐88B, as well as Cu^+^ and Cu^2+^ in Cu_1.8_S, involved in the Fenton/Fenton‐like reactions with the existence of GSH and O_2_^−^ generated in part 2, generating •OH by following formulas:

(2) $$O_{2}+{\mathrm{Cu}}^{+}/{\mathrm{Fe}}^{2+}\to •O_{2}^{-}+{\mathrm{Cu}}^{2+}/{\mathrm{Fe}}^{3+}$$

(3) $$\mathrm{GSH}+•O_{2}^{-}\to {\mathrm{GS}}^{-}+•O_{2}H$$

(4) $$2GS^{-}+2Cu^{2+}/Fe^{3+}\to \mathrm{GSSG}+2Cu^{+}/Fe^{2+}$$

(5) $$2\mathrm{GSH}+2•O_{2}H\to \mathrm{GSSG}+2H_{2}O_{2}$$

(6) $$H_{2}O_{2}+{\mathrm{Cu}}^{2+}/{\mathrm{Fe}}^{3+}\to {\mathrm{Cu}}^{+}/{\mathrm{Fe}}^{2+}•O_{2}H+H^{+}$$

(7) $$H_{2}O_{2}+{\mathrm{Cu}}^{+}/{\mathrm{Fe}}^{2+}•O_{2}H\to •\mathrm{OH}+{\mathrm{Cu}}^{+}/{\mathrm{Fe}}^{2+}+O_{2}+H_{2}O$$

(8) $$H_{2}O_{2}+{\mathrm{Cu}}^{+}/{\mathrm{Fe}}^{2+}+H^{+}\to •\mathrm{OH}+{\mathrm{Cu}}^{2+}/{\mathrm{Fe}}^{3+}+H_{2}O$$

The •OH generation was first investigated by detecting methylene blue (MB) degradation. The UV−vis absorbance curves of MB solution are almost unchanged after adding 100 µL of hydrogen peroxide (H_2_O_2_) (4 mM), while the MB absorptions differently decreased after adding MIL‐88B, Cu_1.8_S and MIL‐Cu_1.8_S (Figure 4F), where the Cu_1.8_S concentration in MIL‐Cu_1.8_S was consistent with the Cu_1.8_S group (40 µg mL^−1^), and MIL‐88B was kept consistent with the iron content in MIL‐Cu_1.8_S. The curve of MIL‐Cu_1.8_S significantly decreases within 360 min, indicating sustained •OH generation (Figure 4G).

The ^1^O_2_ generation was further detected by 9,10‐Anthracenediyl‐bis(methylene)dimalonic acid (ABDA). In the absence of laser illumination, no significant absorption decrease was detected in all samples, indicating little ^1^O_2_ generation (Figure 4H; Figure S13, Supporting Information). Whereas, the ΔA/ A_0_ of MIL‐Cu_1.8_S exposed under an 808‐nm laser was decreased over time (Figure 4H), indicating the generation of ^1^O_2_, which contributed to the semiconductor‐metal heterojunction effect. These results suggest that NIR light plays an important role in the conversion of O_2_ to ^1^O_2_, and MIL‐Cu_1.8_S heterojunctions effectively improve this conversion efficiency. As‐prepared MIL‐Cu_1.8_S can utilize H_2_O_2_ and O_2_ to cross‐catalyze the generation of •OH and ^1^O_2_, respectively.

### 2.4 Cellular Uptake and Subcellular Distribution of MIL‐Cu_1.8_S‐TPP/FA

**Figure 5:**
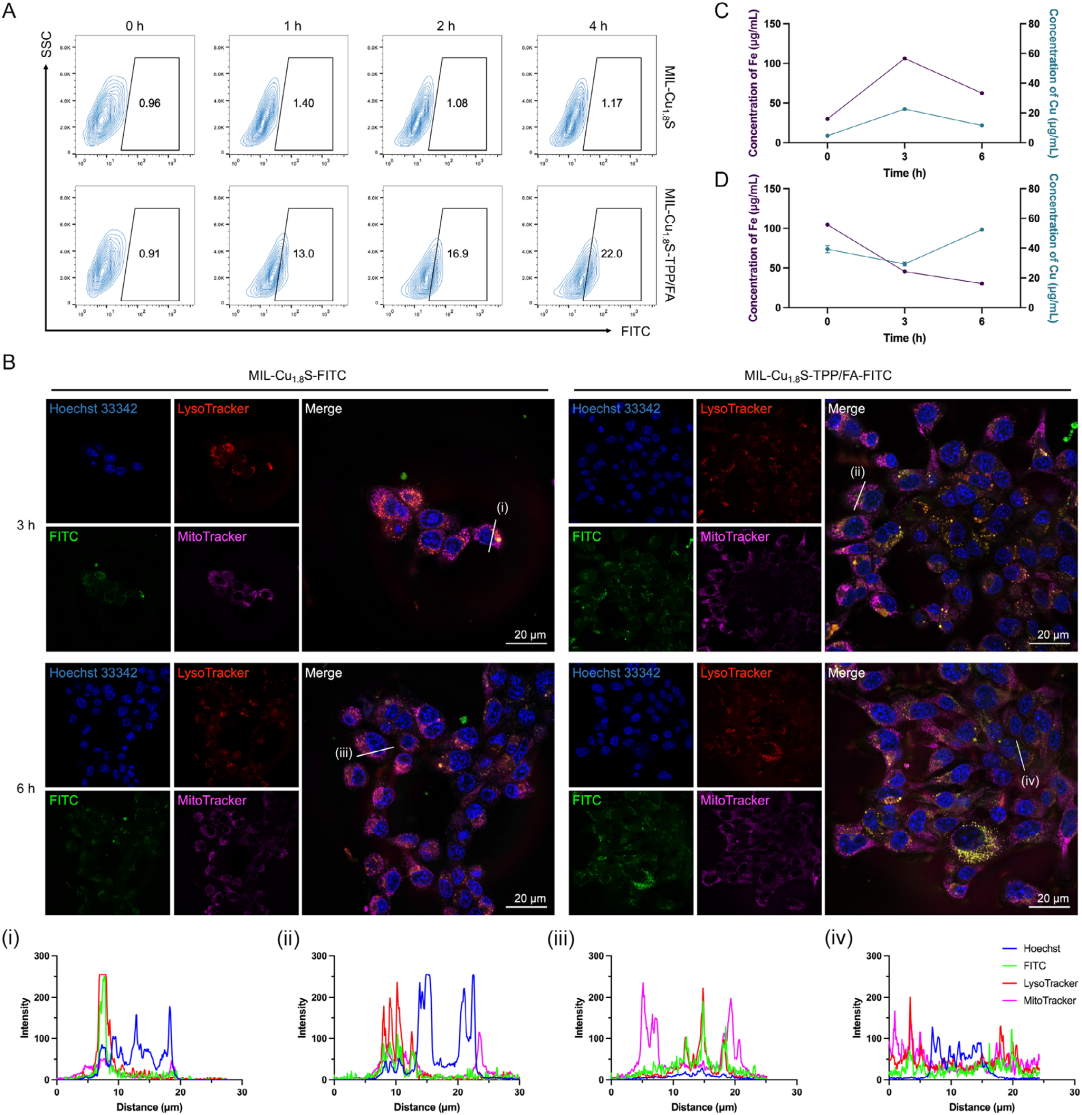
Cellular internalization of MIL‐Cu_1.8_S and MIL‐Cu_1.8_S‐TPP/FA. A) Cell uptake of MIL‐Cu_1.8_S and MIL‐Cu_1.8_S‐TPP/FA detected by Flow cytometry. B) CLSM images of 4T1 cells after 3‐h or 6‐h co‐incubation with MIL‐Cu_1.8_S‐FITC or MIL‐Cu_1.8_S‐TPP/FA/FITC. (i–iv) Linear section (white line) fluorescence intensity profile of Hoechst 33342, FITC, LysoTracker, and MitoTracker in (B). Concentration of iron and copper in C) lysosome and D) mitochondria.

Before investigating the tumor therapeutic effects and mechanisms of MIL‐Cu_1.8_S‐TPP/FA, we determined its cellular uptake. Taking advantage of the strong affinity between Cu and sulfhydryl groups, the Cu_1.8_S nanodots on MIL‐Cu_1.8_S were fluorescently labeled with HS‐FITC to construct MIL‐Cu_1.8_S‐TPP/FA/FITC. Flow cytometry analysis showed a significantly higher percentage of FITC‐positive cells in MIL‐Cu_1.8_S‐TPP/FA/FITC group (22% after 4‐h co‐incubation) compared to MIL‐Cu_1.8_S without the surface modification (1.17%), indicating that the surface modification of FA confers higher cellular uptake of nanoparticles (**Figure** **5**A).

Furthermore, the subcellular localization of MIL‐Cu_1.8_S and MIL‐Cu_1.8_S‐TPP/FA was compared by CLSM. Prior to this, we validated the pH‐responsive dissociation behavior of Cu_1.8_S nanodots from MIL‐Cu_1.8_S‐TPP/FA in an acidic environment mimicking lysosome due to the acid‐sensitive degradation of MIL‐88B. As shown in Figure S14 (Supporting Information), incubation of MIL‐Cu_1.8_S‐TPP/FA in pH‐7.4 medium for several hours caused no observable changes in the UV−vis absorption spectrum of the supernatant after centrifugation (14 000 rpm, 10 min), whereas incubation under acidic conditions (pH 5.7) resulted in detectable Cu_1.8_S absorption in the supernatant, confirming that the nanodots dissociate upon MIL‐88B degradation under lysosomal‐mimicking conditions. CLSM imaging revealed significantly stronger intracellular green fluorescence in MIL‐Cu_1.8_S‐TPP/FA/FITC‐treated cells compared to the MIL‐Cu_1.8_S‐FITC group, further verifying the FA‐enhanced uptake (Figure 5B). After 3‐h co‐incubation with MIL‐Cu_1.8_S‐TPP/FA/FITC, most green fluorescently labeled MIL‐Cu_1.8_S‐TPP/FA/FITC colocalized with red lysosomes, indicating initial accumulation in lysosomes. By 6 h, the co‐localization with lysosomes was reduced, and the fluorescence increasingly overlapped with magenta‐colored mitochondria, suggesting lysosomal escape and mitochondrial translocation of the Cu_1.8_S‐TPP/FA/FITC. These dynamic changes were further visualized in fluorescence intensity line profiles of representative cells (Figure 5B and i–iv). In contrast, MIL‐Cu_1.8_S‐FITC without TPP modification did not exhibit significant lysosomal escape or mitochondrial accumulation at 6 h, with green fluorescence remaining largely confined within lysosomes. This comparison highlights the essential role of TPP functionalization in facilitating mitochondrial targeting of Cu_1.8_S nanodots following lysosomal release.

To validate the subcellular distribution of metal ions, lysosomes, and mitochondria were isolated from 4T1 cells after MIL‐Cu_1.8_S‐TPP/FA treatment at different time points and analyzed by ICP‐OES. At 3 h post‐treatment, a significant accumulation of Fe and Cu was observed in lysosomes due to the endocytosis of MIL‐Cu_1.8_S‐TPP/FA (Figure 5C). At 6 h, lysosomal metal content declined sharply, whereas mitochondrial Cu showed a moderate increase, and mitochondrial Fe continued to decrease (Figure 5D). These results suggest that Cu ions translocate from lysosomes to mitochondria, where TPP‐mediated targeting facilitates their accumulation. The persistent decline in mitochondrial Fe may reflect Fe‐S cluster disruption and iron efflux, a hallmark of copper‐induced mitochondrial dysfunction.

To further distinguish the localization of the MIL‐88B carrier from that of the Cu_1.8_S nanodots, we constructed a control probe, FITC@MIL‐Cu_1.8_S‐TPP/FA, in which the green fluorescence FITC molecules were encapsulated within MIL‐88B rather than bound to Cu_1.8_S. CLSM images showed that, even after 6 h of incubation, green fluorescence from FITC@MIL‐Cu_1.8_S‐TPP/FA largely co‐localized with lysosomes (Figure S15, Supporting Information), indicating that the MIL‐88B framework remained confined to lysosomes. Together, these results highlight the spatiotemporal dynamics of MIL‐Cu_1.8_S‐TPP/FA trafficking and metal redistribution, which underlie the transition from lysosome‐dependent ion release to mitochondria‐centered cuproptosis activation.

### 2.5 In Vitro Anti‐Tumor Effect and Mediation of Cellular Ferroptosis and Cuproptosis

**Figure 6:**
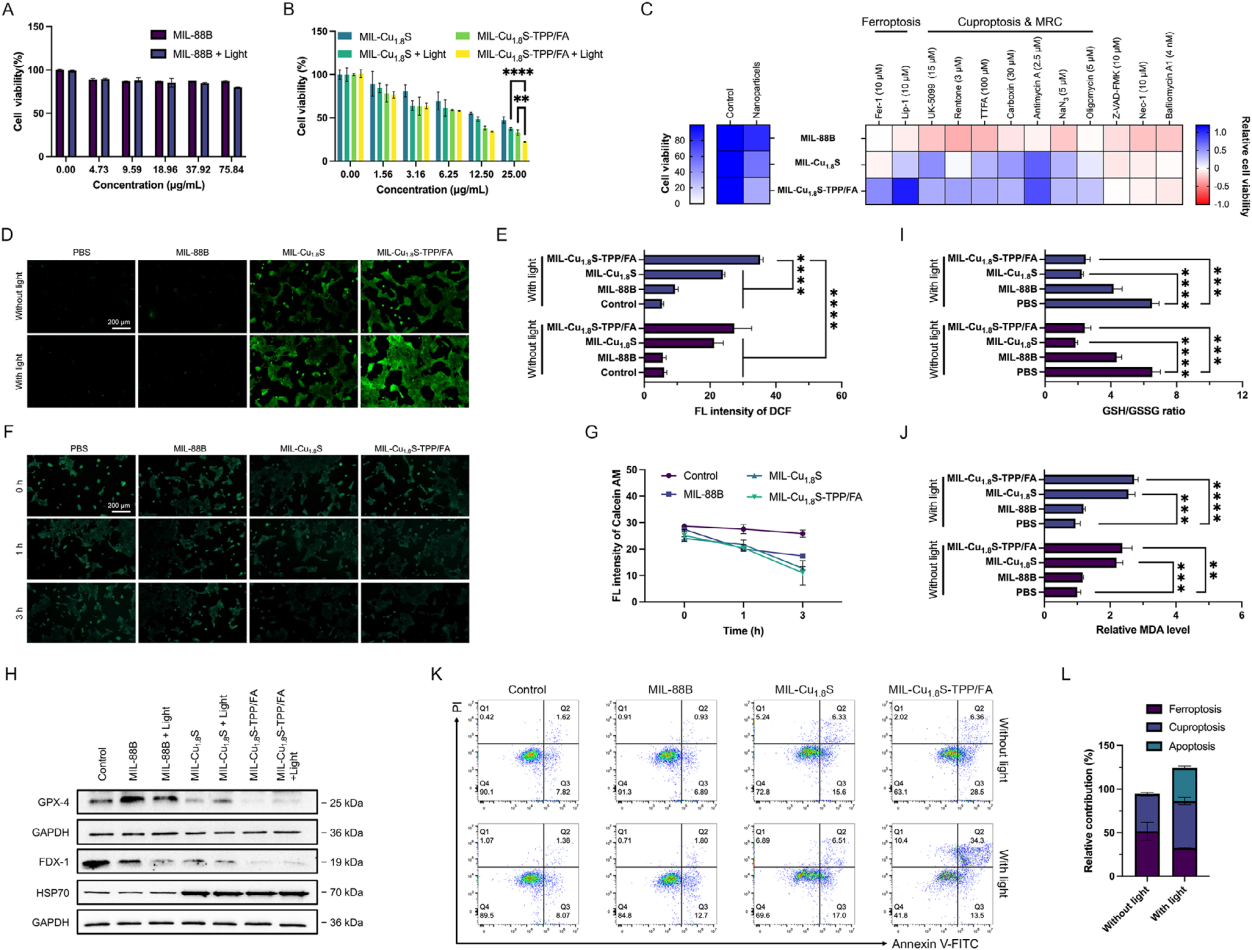
In vitro ferroptotic and cuproptotic anti‐cancer effect and of MIL‐Cu_1.8_S‐TPP/FA. A) Cell viability of MIL‐88B with/without light in 4T1 cells. B) Cell viability of MIL‐Cu_1.8_S and MIL‐Cu_1.8_S‐TPP/FA with/without light in 4T1 cells. C) Cell viability of 4T1 cells pretreated with various inhibitors and then treated with MIL‐88B, MIL‐Cu_1.8_S, and MIL‐Cu_1.8_S‐TPP/FA. The relative cell viability is relative to cell viability with the treatment of the same inhibitor (n = 3, the bar charts are shown in Figure S17, Supporting Information). D) In vitro ROS generation observed by CLSM images and E) their respective fluorescence intensity analysis. F) Intracellular labile iron after treatments of MIL‐88B, MIL‐Cu_1.8_S, or MIL‐Cu_1.8_S‐TPP/FA for different times detected by Calcein‐AM and observed by fluorescence microscope images and G) their respective fluorescence intensity analysis. H) Western blot of ferroptosis‐related protein GPX4 and cuproptosis‐related proteins FDX‐1 and HSP70 treated with MIL‐88B, MIL‐Cu_1.8_S or MIL‐Cu_1.8_S‐TPP/FA with/without NIR light irradiation. I) Measurement of GSH/GSSH ratio and J) relative MDA levels in 4T1 cells after treatment with different samples. K) Flow cytometry analysis of 4T1 cells treated with MIL‐88B, MIL‐Cu_1.8_S, or MIL‐Cu_1.8_S‐TPP/FA with/without NIR light irradiation. L) The relative contribution of ferroptosis, cuproptosis, and apoptosis. ** *P* < 0.01, *** *P* < 0.001, **** *P* < 0.0001.

The anti‐cancer effects of MIL‐Cu_1.8_S‐TPP/FA were evaluated by CCK‐8. The cell viability of 4T1 cells after 24 h treatment of MIL‐88B with/without NIR light irradiation was higher than 80% (**Figure** **6**A). Without NIR light irradiation, MIL‐Cu_1.8_S and MIL‐Cu_1.8_S‐TPP/FA presented considerable toxicities, where the cell viabilities were 47.54% and 33.02% respectively at the Cu_1.8_S concentration of 25 µg mL^−1^ contained in nanocomposites (Figure 6B), while Cu_1.8_S nanodots at equal co‐culture concentration presented cell viability of 80.79% (Figure 2B). This enhancement can be attributed not only to the higher cellular internalization due to the size and roughness of MIL‐Cu_1.8_S but also to the synergy effect between iron and copper ions. Notably, the cell toxicities of groups of Cu_1.8_S, MIL‐Cu_1.8_S, and MIL‐Cu_1.8_S‐TPP/FA with light are also attributed to the photothermal effect due to the existence of Cu_1.8_S. With NIR light, the cell viabilities of MIL‐Cu_1.8_S and MIL‐Cu_1.8_S‐TPP/FA groups further decreased to 37.43% and 22.35% respectively at the same concentration (Figure 6B). Compared to MIL‐Cu_1.8_S, MIL‐Cu_1.8_S‐TPP/FA showed higher toxicity, resulting from better cellular uptake and subcellular targeting. Similar trends were also observed in the MB‐MDA‐231 cell line (Figure S16, Supporting Information).

Based on the knowledge of metal‐regulating cell death and ROS‐generating effect of MIL‐Cu_1.8_S that was proven in a simulated environment,^[^ ^22^ ^]^ the anti‐cancer effects of MIL‐Cu_1.8_S‐TPP/FA are mainly attributed to inducing cancer cell ferroptosis and cuproptosis due to the introduction and release of a large amount of iron and copper ions inside cells over a short period time under lysosomal acid and NIR light stimulation. As shown in Figure 6C and Figure S17 (Supporting Information), the typical inhibitors of ferroptosis (ferrostatin‐1 (Fer‐1) and liproxstatin‐1 (Lip‐1)), cuproptosis (UK5099), and mitochondrial respiratory chain (MRC) complexes (rentone, 2‐thenoyltrifluoroacetone (TTFA), carboxin, antimycin A, NaN_3_, and oligomycin) rescued killing by MIL‐Cu_1.8_S‐TPP/FA (Cu_1.8_S concentration of 25 µg mL^−1^), while the inhibitors of apoptosis, necrosis and autophagy (Z‐VAD‐FMK, necrostatin‐1 (Nec‐1) and bafilomycin A1, respectively) could not rescue cellular killing, indicating the cell death induced by MIL‐Cu_1.8_S‐TPP/FA was closely related to ferroptosis and cuproptosis. Notably, the viability of 4T1 cells treated with MIL‐88B at concentrations below 75 µg mL^−1^ exhibit few responses to ferroptosis inhibitor Fer‐1 because the presence of iron ions mainly contributes to the disappearance of initiating LPO that is at a relatively low level.^[^ ^23^ ^]^ Whereas, the significant rescue of cell viability in the MIL‐Cu_1.8_S‐TPP/FA group demonstrates that intracellular LPO level increases after treatment of MIL‐Cu_1.8_S‐TPP/FA, indicating copper has an essential function in ferroptosis and is distinct from that of iron. To further investigate the anti‐cancer mechanism of MIL‐Cu_1.8_S‐TPP/FA, we detected the intracellular ROS level by 2′,7′‐dichlorodihydrofluorescein diacetate (DCFH_2_‐DA), as the fluorescent ROS indicator. ROS are biological molecules that participate in ferroptosis and cuproptosis.^[^ ^2^, ^24^ ^]^ As depicted in Figure 6D, E, the MIL‐Cu_1.8_S‐TPP/FA under 808‐nm laser exposure for 10 min in 4T1 cells exhibited a significantly higher green fluorescence intensity than the control group, indicating more ^1^O_2_ and •OH generation via the semiconductor‐metal heterojunction catalysis and the Fenton‐/Fenton‐like reaction, respectively. NIR light was necessary for ^1^O_2_ generation but also improved the •OH generation due to introducing a higher local temperature (i.e., photothermal‐enhanced chemodynamic therapy).^[^ ^25^ ^]^ The fluorescence microscope images show a better visual display of intracellular labile iron that was detected by calcein‐acetoxymethyl ester (Calcein‐AM).^[^ ^26^ ^]^ The green fluorescence of Calcein‐AM diminished to varying degrees as the cells were co‐incubated with MIL‐88B, MIL‐Cu_1.8_S, and MIL‐Cu_1.8_S‐TPP/FA for longer periods of time (Figure 6F,G), indicating increased labile iron levels. Whereas the more remarkable decrease in fluorescence intensity in MIL‐Cu_1.8_S and MIL‐Cu_1.8_S‐TPP/FA groups can be attributed to the increased uptake rate due to the higher surface roughness of MIL‐Cu_1.8_S compared to MIL‐88B.^[^ ^27^ ^]^ Similar to the results of cell viability, MIL‐88B in the low concentration (below 75 µg mL^−1^) neither induced significant cytoxicity nor decreased the expression level of GPX4 (Figure 6H). Whereas, expression of GPX4 in 4T1 cells after treatments of MIL‐Cu_1.8_S or MIL‐Cu_1.8_S‐TPP/FA with/without NIR light irradiated significantly decreased because large amounts of copper ions were involved in the transformation of GSH to GSSG, inactivating GPX4.^[^ ^5^, ^28^ ^]^ Additionally, GPX4 has been reported a autophagic degradation,^[^ ^29^ ^]^ resulting in the low expression of it in MIL‐Cu_1.8_S or MIL‐Cu_1.8_S‐TPP/FA groups. Meanwhile, the significant reduction in the intracellular GSH/GSSG ratio after 4T1 cells treated with MIL‐Cu_1.8_S‐TPP/FA indicates the catalytic effect of copper and iron ions in the MIL‐Cu_1.8_S‐TPP/FA, as well as the production of ROS, whereby the cells consume more GSH to mitigate the oxidation level (Figure 6I). The damage to cell membrane was measured by assessing the MDA levels, a product of LPO formed through ROS mediated by released copper and iron ions. The MDA level in 4T1 cells treated with MIL‐Cu_1.8_S‐TPP/FA was significantly increased (Figure 6J), indicating LPO proceedings.

FDX‐1, as a key factor of the TCA cycle, was down‐regulated by MIL‐Cu_1.8_S or MIL‐Cu_1.8_S‐TPP/FA owing to the existence of mitochondria‐targeting Cu_1.8_S.^[^ ^30^ ^]^ Combining these copper‐induced cellular effect, including down‐regulation of GPX4 and FDX‐1, and effect on MRC, as well as intracellular photothermal conversion, the expression of HSP70 dramatically increased (Figure 6H; Figure S18, Supporting Information), reflecting acute proteotoxic stress.^[^ ^3a^ ^]^ Considering the close relationship between copper ions and ferroptosis and cuprotosis, we analyzed the cross‐talk between ferroptosis and cuproptosis regulators for breast cancer from GEO (GSE15852). Principal component analysis (PCA) illustrated a relatively evident distinction that existed in the 2 clusters (Figure S19, Supporting Information). Figure S20 (Supporting Information) displays strong correlations between ferroptosis and cuproptosis regulators, which corroborates the rationale for employing copper and iron ions cross‐induced cancer cell death strategy. Furthermore, the synergistic effect was also investigated using Annexin V‐FITC and PI staining by flow cytometry. The 4T1 cells with MIL‐Cu_1.8_S‐TPP/FA treated showed an apoptosis‐like cell death. The proportion of viable 4T1 cells was more than 85% in the presence of NIR light‐irradiated control and MIL‐88B groups, while the viability of 4T1 cells in MIL‐Cu_1.8_S and MIL‐Cu_1.8_S‐TPP/FA groups were 72.8% and 63.1%, respectively. In the presence of NIR light irradiation, the viability of 4T1 cells in these two groups decreased to 69.6% and 41.8%, respectively (Figure 6K). Due to the difference between the ferroptosis or cuproptotic pathways and apoptosis, there was some discrepancy between the flow cytometry results and the cytotoxicity results of CCK‐8 assay, and 808 nm light, in particular, showed more effects in the annexin‐V/PI‐based results, suggesting that photothermal therapy may contribute more to apoptosis.

Subsequently, to elucidate the relative contributions of different cell death pathways induced by MIL‐Cu_1.8_S‐TPP/FA, quantitative inhibitor‐based analyses were conducted under conditions with and without NIR irradiation (Figure S21, Supporting Information). In the absence of photothermal activation, ferroptosis and cuproptosis accounted for 51.5% and 42.4% of the total cytotoxicity, respectively, while apoptosis contributed only 0.7%. Upon NIR exposure, however, the contribution of cuproptosis markedly increased to 53.9%, accompanied by a significant elevation in apoptosis to 38.0%, while the ferroptotic contribution decreased to 32.4% (Figure 6L). These findings indicate that photothermal stimulation not only enhances cuproptosis but also activates apoptotic pathways, partially shifting the cell death modality away from ferroptosis. This transition correlates with the elevated intracellular ROS levels observed under photothermal conditions, as confirmed by DCFH‐DA fluorescence staining (Figure 6D). Notably, the GSH/GSSG ratio remained largely unchanged, suggesting that the ferroptosis pathway was not further enhanced due to insufficient glutathione depletion. Mechanistically, this shift can be attributed to the photothermal‐responsive property of the MIL‐Cu_1.8_S‐TPP/FA. Under NIR irradiation, local heating accelerates the dissociation of Cu_1.8_S nanodots, particularly within mitochondria owing to the TPP‐mediated subcellular targeting. Overall, photothermal activation not only increases overall cytotoxicity but also rewires the dominant death pathway toward mitochondrial cuproptosis, highlighting the importance of spatially targeted copper delivery in enhancing therapeutic efficacy.

### 2.6 In Vivo Distribution of MIL‐Cu_1.8_S‐TPP/FA and Multi‐Model Tumor Therapy

**Figure 7:**
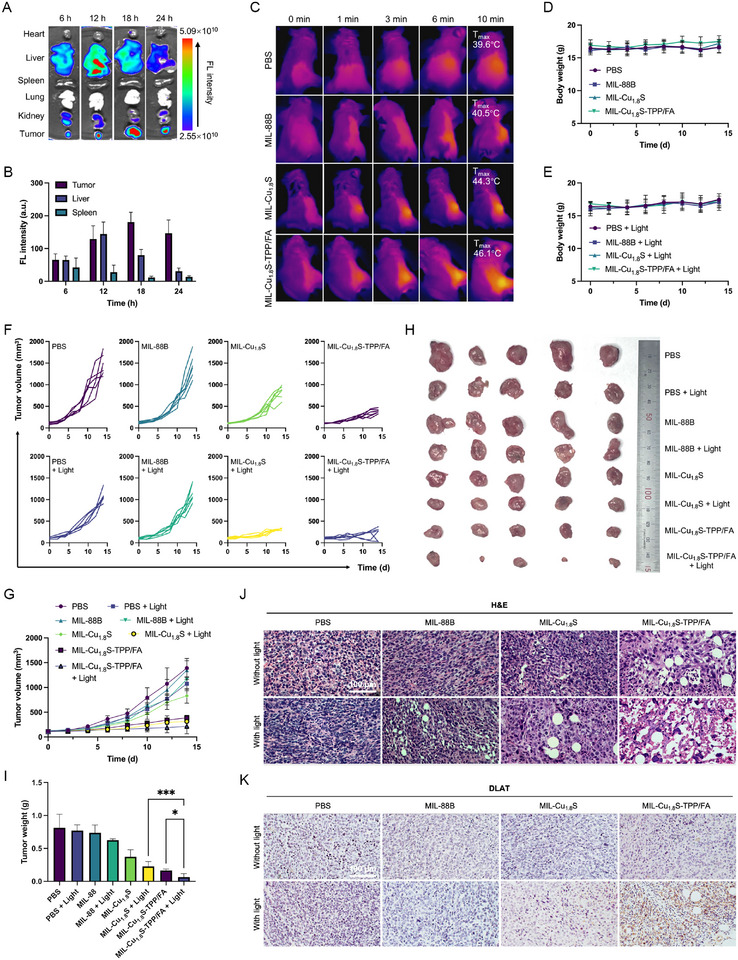
Biodistribution and in vivo anti‐tumor effect of MIL‐Cu_1.8_S‐TPP/FA. A) Fluorescence images of major organs and tumors of mice treated with MIL‐Cu_1.8_S‐TPP/FA at different time points after tail‐vein injection. B) Average fluorescence intensity of tumor, liver, and spleen at different time points after tail‐vein injection of MIL‐Cu_1.8_S‐TPP/FA. C) Thermal infrared imaging of in vivo photothermal performance. Body weight of mice treated by PBS, MIL‐88B, MIL‐Cu_1.8_S, and MIL‐Cu1.8S‐TPP/FA D) without NIR light irradiation and E) with NIR light irradiation, respectively. F) The individual and G) mean tumor volume of mice with treated PBS, MIL‐88B, MIL‐Cu_1.8_S, and MIL‐Cu_1.8_S‐TPP/FA with/without NIR light irradiation. H) Optical image of tumors collected from mice 14 d after treatment. I) Tumor weight of mice 14 d after different treatment. J) H&E staining images of major organs and tumors after 14 d treatment with PBS, MIL‐88B, MIL‐Cu1.8S, and MIL‐Cu1.8S‐TPP/FA with/without NIR light irradiation. K) Immunohistochemical analysis of DLAT of 4T1 tumor tissue with various treatments. * *P* < 0.05, *** *P* < 0.001.

To further explore the tumor inhibition ability of MIL‐Cu_1.8_S‐TPP/FA in vivo, the Balb/c female mice were inoculated with 4T1 cells to subcutaneously grow tumors in their right flank. When the tumors reached ≈80 mm^3^, the tumor‐bearing mice were employed to evaluate the tumor suppression efficacy of MIL‐Cu_1.8_S‐TPP/FA. Before treating mice, the hemolysis of MIL‐Cu_1.8_S‐TPP/FA was tested. It was observed that the hemolysis rate of MIL‐Cu_1.8_S‐TPP/FA was less than 5%, indicating the good blood compatibility of MIL‐Cu_1.8_S‐TPP/FA (Figure S22, Supporting Information). Subsequently, tumor enrichment and major organ excretion of MIL‐Cu_1.8_S‐TPP/FA in mice were investigated for designing the administration. After intravenous injections of fluorescein isothiocyanate (FITC)‐labeled MIL‐Cu_1.8_S‐TPP/FA at different time points (6, 12, 18, and 24 h), the major organs (heart, liver, spleen, lung, and kidney) and tumors of mice were collected and observed by an animal bio‐luminescence system (ABL‐X6, Tanon, Shanghai, China). Benefiting from the FA modification, the fluorescence intensity of the tumor treated with MIL‐Cu_1.8_S‐TPP/FA was higher than that of the tumor treated with MIL‐Cu_1.8_S at 12 h and 18 h after injection (Figure S23, Supporting Information), indicating higher tumor enrichment and longer tumor retention. As shown in **Figure** **7**A, the fluorescence intensities are high in the liver and kidney at 6 and 12 h post injection and obviously reduce at 18 and 24 h post injection, indicating MIL‐Cu_1.8_S‐TPP/FA can be metabolized and excreted through feces and urine. In contrast, the fluorescence in tumors continually increases at 6 to 18 h after injection and decreases at 24 h post injection (Figure 7B). Thus, the 4T1 tumor‐bearing mice in light‐irradiation groups were given 808 nm laser (1.5 W cm^−2^) administration at the tumor site for 10 min after 18 h post intravenously injection. Tumor‐bearing mice were randomly separated into eight groups (n = 5), i.e., PBS, MIL‐88B, MIL‐Cu_1.8_S, and MIL‐Cu_1.8_S‐TPP/FA and their respective groups with laser irradiation, i.e., PBS + Light, MIL‐88B + Light, MIL‐Cu_1.8_S + Light, and MIL‐Cu_1.8_S‐TPP/FA + Light. The mentioned protocols were applied on day 0 and day 7. Figure 7C displays the in vivo photothermal effect of the mentioned four samples. Under an 808 nm NIR light irradiation, the tumor sites of mice treated with MIL‐Cu_1.8_S and MIL‐Cu_1.8_S‐TPP/FA were heated to 44.3 and 46.1 °C, respectively, witnessing the photothermal effect of MIL‐Cu_1.8_S and better tumor enrichment of tumor‐targeting‐modified MIL‐Cu_1.8_S‐TPP/FA. In contrast, under the same condition, the temperature of the tumor sites of mice treated with PBS and MIL‐88B were maintained below 40.5 °C. All groups show no apparent body weight change during the 14 d of the observation period (Figure 7D,E). Additionally, hematoxylin‐eosin (H&E) staining images of major organs display no obvious inflammation or other pathological change in groups, showing the excellent biocompatibility of samples (Figure S24, Supporting Information). The individual tumor volume progression, mean tumor volume, representative optical tumor images, and tumor weight at 14 days post‐treatment are presented in Figure 7F−I, respectively. Among all treatment groups, the MIL‐Cu_1.8_S‐TPP/FA + Light group exhibited the most significant tumor suppression, with markedly reduced tumor volume, weight, and visible size. The tumor growth rate in the MIL‐Cu_1.8_S‐TPP/FA + Light group was only 97.44%, significantly lower than that of the MIL‐Cu_1.8_S‐TPP/FA group (261.69%), the MIL‐Cu_1.8_S + Light group (183.95%), and the PBS negative control group (917.89%). These results highlight the synergistic antitumor effects resulting from photothermal therapy and dual‐targeting modifications. H&E staining revealed extensive nuclear atypia and cytoplasmic disintegration in tumor tissues from the MIL‐Cu_1.8_S‐TPP/FA + Light group (Figure 7J), indicating severe tumor cell damage. To further investigate the mechanism of copper‐induced cytotoxicity, immunohistochemical staining was performed to assess the expression and aggregation of DLAT (Figure 7K). Notably, the MIL‐Cu_1.8_S‐TPP/FA + Light group exhibited the most substantial DLAT aggregation among all groups. Collectively, these in vivo results demonstrate that MIL‐Cu_1.8_S‐TPP/FA combined with photothermal activation elicits robust anti‐tumor effects through a multi‐modal mechanism involving photothermal ablation, ferroptosis, and cuproptosis.

## 3 Conclusion

In summary, this study demonstrates that mitochondria‐targeted modification enhances the cytotoxicity of biocompatible Cu_1.8_S nanodots by inducing cellular cuproptosis. Building on this insight, we employed a secondary growth method to construct MIL‐Cu_1.8_S by growing Cu_1.8_S nanodots on the surface of MIL‐88B. The resulting MIL‐Cu_1.8_S exhibited potent POD‐like catalytic activity, enhancing •OH generation and promoting the conversion of O_2_ into ^1^O_2_. After being modified with FA and TPP on the surface of Cu_1.8_S embedded MIL‐88B, the prepared MIL‐Cu_1.8_S‐TPP/FA exhibited significantly higher in vitro cellular uptake and mitochondrial subcellular‐targeting capabilities, as well as in vivo tumor enrichment. Additionally, the pH‐responsive and photothermal properties of MIL‐Cu_1.8_S‐TPP/FA facilitated the controlled release of iron and copper ions, triggering ferroptosis and cuproptosis via GSH depletion, GPX4 inactivation, and FDX‐1 downregulation in tumor cells. The MIL‐Cu_1.8_S‐TPP/FA demonstrated robust anti‐tumor efficacy both in vitro and in vivo. Importantly, this approach holds potential for broader application across different tumor types, offering a foundation for advancing the therapeutic and clinical translation of metal‐based nanomaterials in cancer therapy.

## Conflict of Interest

The authors declare no conflict of interest.

## Supporting information



Supporting Information

## Data Availability

Research data are not shared.
