# Supplementary material for: A Mitochondria‐Targeted Nanozyme Platform for Multi‐Pathway Tumor Therapy via Ferroptosis and Cuproptosis Regulation
Source: Adv Sci (Weinh). 2025 Jun 25;12(36):e17616. doi: 10.1002/advs.202417616 (PMC12462976; doi:10.1002/advs.202417616)
Supplement: Supplementary file 1 — Supporting Information [file ADVS-12-e17616-s001.docx]

Supporting Information

A Mitochondria-Targeted Nanozyme Platform for Multi-Pathway Tumor Therapy via Ferroptosis and Cuproptosis Regulation

Chenguang Liu*, Lingxiao Guo, Yuying Cheng, Jingjie Gao, Hanling Pan, Jiayi Zhu, Danting Li, Liqing Jiao and Caiyun Fu*

**1. Materials and methods**

**1.1. Materials**

Iron chloride dihydrate (FeCl_3_•6H_2_O), Copper chloride dihydrate (CuCl_2_•2H_2_O), sodium sulfide nonahydrate (Na_2_S•9H_2_O), Sodium hydroxide (NaOH), potassium phosphate monobasic (KH_2_PO_4_), potassium phosphate dibasic (K_2_HPO_4_), sodium phosphate dibasic (Na_2_HPO_4_), methylene blue (MB) and 20,70-dichlorodihydrofluorescein diacetate (H_2_-DCFDA) were obtained from Macklin (Shanghai, China). 9,10-Anthracenediyl-bis(methylene)dimalonic acid (ABDA) and Calcein-AM were purchased from MedChemExpress (Shanghai, China). Poly-(vinylpyrrolidone) (PVP) K15 (viscosity average molecular 10,000 Da) was obtained from Tokyo Chemical Industry Co., Ltd (Tokyo, Japan). HS-PEG-FITC and HS-PEG-TPP (viscosity average molecular 5000 Da) were obtained from Xinqiao Biotechnology Co., Ltd. (Hangzhou, China). Radio immunoprecipitation assay (RIPA) lysis buffer, phenylmethanesulfonylfluoride (PMSF) and enhanced chemiluminescence (ECL) assay were purchased from Solarbio Science & Technology Co. Ltd. (Beijing, China).

**1.2. Characterization**

The morphology of MIL-Cu_1.8_S were characterized by scanning electron microscopy (SEM, JSM-5610LV, JEOL, Tokyo, Japan) and transmission electron microscopy (TEM, FEI talos F200x G2, Thermo Fisher Scientific, Waltham, U.S.) with EDS super-X, respectively. UV−vis absorption was recorded on a UV-1800 spectrophotometer (Shimadzu, Tokyo, Japan). Zeta potential and size distribution of samples in water were determined by using Zetasizer Nano ZS90 (Malvern Panalytical, Malvern, U.K.). The weights (%) of Fe and Cu in MIL-Cu_1.8_S were determined by an inductively coupled plasma optical emission spectrometer (ICP-OES, 5110, Agilent, Palo Alto, U.S.). The characteristic basal reflection patterns of samples were evaluated using X-ray diffraction (XRD, X'Pert3 Powder, Panalytical, Alemlo, Netherlands) analysis in two Theta range of 5-20 ° (Cu Ka radiation, 40 kV, 40 mA). The in-depth chemical composition and valence state of Copper in MIL-Cu_1.8_S was analyzed by X-ray photoelectron spectroscopy (XPS, K-Alpha, Thermo Fisher Scientific). The functional group confirmations were made by recording the FTIR spectra on a Nicolet iS20 spectrometer (Thermo Fisher Scientific) using a dried KBr pellet method.

**1.3. Preparation and surface modification of Cu_1.8_S nanodots**

17 mg of CuCl_2_•2H_2_O and 3 g of PVP were dissolved in 100 mL of ultrapure water. The mixture was put under vigorous agitation in an oil bath at 90 °C for 10 min. Subsequently, a 1 M Na_2_S solution (100 μL) was added to the above mixture. Further, the mixture was maintained at 90 °C and stirred for another 20 min until the mixture turned to dark green, followed by a rapid cooldown in an ice-cold water bath.

To prepare Cu_1.8_S-PEG-TPP, 2 mL of as-obtained Cu_1.8_S nanodots (2.5 mg mL^−1^) were added to ultrapure water with 0.5 mg of HS-PEG-TPP. After the mixture was kept stirring overnight at 40 °C, Cu_1.8_S-PEG-TPP was collected and wash by ultrapure water twice to remove unbound HS-PEG-TPP.

**1.4. Preparation of MIL-88B, MIL-Cu_1.8_S and MIL-Cu_1.8_S-TPP/FA**

MIL-88B seeds were synthesized by mixing 13.5 mg of FeCl_3_•6H_2_O and 9 mg of BDC-NH_2_ in 10 mL of ethanol with stirring at 60 °C for 0.5 h. For secondary growth, another mixture of FeCl_3_•6H_2_O and BDC-NH_2_ was dropwise added to above MIL-88B seeds suspension with stirring at 40 °C for 0.5 h. In the case of MIL-Cu_1.8_S, 2 mg of as-prepared Cu_1.8_S nanodots were mixed during the secondary growth process. MIL-Cu_1.8_S was collected by centrifuged at 13000 rpm for 10 min and washed by ultrapure water for three times. For further surface-modification, 10 mg of MIL-Cu_1.8_S was resuspend in 8 mL of ultrapure water with 0.5 mg of HS-PEG-TPP and 0.5 mg of HS-PEG-FA. After the mixture was kept stirring overnight at room temperature, MIL-Cu_1.8_S-TPP/FA was collected and wash by ultrapure water and EtOH twice.

**1.5. Extracellular Photothermal Conversion and ROS Generation of MIL-Cu_1.8_S**

1 mL of the aqueous suspension of Cu_1.8_S (50 μg mL^−1^) was placed in a quartz cell and irradiated using an 808-nm NIR laser at gradient power densities (0.5, 1.0, 1.5, 2.0 W cm^−2^) for 10 min. The temperature of the suspension was measured every 30 s. Similarly, MIL-Cu_1.8_S (Cu_1.8_S concentration of 50 μg mL^−1^) were also administered at a power density of 1.5 W cm^−2^. Control groups, including ultrapure water and MIL-88B suspension groups, were treated under the same conditions. The photothermal conversion efficiencies (*η*) of Cu_1.8_S and MIL-Cu_1.8_S with different Cu_1.8_S proportion were calculated according to the following equation:

$ƞ=\frac{hA(\Delta T_{max,mix}-\Delta T_{max,H_{2}O})}{I(1-{10}^{-A_{\lambda}})}$(1)

where *h* is the heat transfer coefficient, *A* is the surface area of the container, Δ*T*_max,mix_ and Δ*T*_max,H2O_ are the temperature change of the samples dispersion and water at the maximum steady-state temperature, respectively, *I* is the laser power, and *A_𝜆_* is the absorbance of samples at 808 nm.

Methylene blue (MB) and ABDA were employed to detect •OH and ^1^O_2_, respectively. MIL-88B, Cu_1.8_S and MIL-Cu_1.8_S were resuspended in PBS with different pH value, respectively. The concentration of Cu_1.8_S in MIL-Cu_1.8_S were consistent with the Cu_1.8_S group (40 μg mL^−1^), and MIL-88B was kept consistent with the iron content in MIL-Cu_1.8_S. Then, 10 mM H_2_O_2_ and 10 μg mL^−1^ MB were mixed. Immediately, •OH-triggered MB degradation was indicated by absorbance change at gradient time intervals. Similarly, ^1^O_2_ was detected by measuring the absorbance decrease of ABDA (100 μM). For the NIR light irrdiration groups, the mixtrure were irradiated using an 808-nm laser at 1.5 W cm^−2^ for different time.

**1.6. Density functional theory (DFT) calculation method**

The spin-polarized density functional theory (DFT) calculations were carried out in the Vienna ab initio simulation package (VASP) based on the plane-wave basis sets with the projector augmented-wave method. The exchange-correlation potential was treated by using a generalized gradient approximation (GGA) with the Perdew-Burke-Ernzerhof (PBE) parametrization. The value of the effective Hubbard U was set as 3.0 eV for Fe. The van der Waals correction of Grimme’s DFT-D3 model was adopted. The energy cutoff was set to be 450 eV. The Brillouin-zone integration was sampled with a single Gamma point (1 × 1 × 1). The structures were fully relaxed until the maximum force on each atom was less than 0.03 eV/Å, and the energy convergent standard was 10^-5^ eV. The Gibbs free energies of the intermediates were calculated using the following expression:

$\Delta G=\Delta E+\Delta E_{ZPE}+\Delta H_{0\to T}-T\Delta S$ (2)

where *ΔE* denotes the change in electronic energy obtained from DFT, *ΔE_ZPE_*, *ΔH_0→T_* and ΔS are the changes of the zero-point energy, the enthalpy and entropy at standard conditions (T= 298 K and at potential vs NHE).

**1.7. Cell culture**

Breast cancer cell line (4T1 and MDA-MB-231) was purchased from American Type Culture Collection (ATCC, Manassas, USA). Cells were cultivated in the 10% FBS-contained DMEM in a humidified incubator maintained at 37 °C and 5% CO_2_.

**1.8. Cellular uptake**

4T1 cells were cultured and seeded in a laser confocal petri dish. To investigate the cellular uptake and subcellular distribution of nanocomposites, cells were co-incubated with either FITC-labeled MIL-Cu_1.8_S and MIL-Cu_1.8_S-TPP/FA by conjugating HS-FITC with copper (i.e., MIL-Cu_1.8_S-FITC and MIL-Cu_1.8_S-TPP/FA/FITC) or FITC-loaded MIL-Cu_1.8_S (FITC loaded in the pore of MIL-88B, FITC@MIL-Cu_1.8_S) at a concentration of 10 μg mL^−1^ in a medium. The nucleus, lysosome and mitochondria were stained with Hoechst 33342 (10 μg mL^−1^), Lyso-Tracker Red (100 nM) and Mito-Tracker Deep Red (100 nM). The cells were observed by CLSM and analyzed by imageJ.

Additionally, 4T1 cells co-incubated with MIL-Cu_1.8_S-FITC and MIL-Cu_1.8_S-TPP/FA/FITC were collected for detection of flow cytometer (Attune NxT, Thermo Fisher Scientific, Waltham, U.S.). Meanwhile, 4T1 cells treated with MIL-Cu_1.8_S-FITC and MIL-Cu_1.8_S-TPP/FA/FITC were also collected and counted for isolation of mitochondria and lysosome. Specifically, 5×10^6^ 4T1 cells for each group were collected and homogenized on ice. The homogenate was centrifuged at 800 × g for 10 minutes at 4 °C to remove intact cells and nuclei, and the resulting supernatant was collected for organelle isolation. Mitochondria were pelleted by centrifuging the supernatant at 10,000 × g for 15 minutes at 4 °C and then washed once with cold buffer. The post-mitochondrial supernatant was subsequently centrifuged at 20,000 × g for 20 minutes to collect lysosomes. All organelle pellets were gently resuspended for further metal quantification by ICP-OES after acid digestion.

**1.9. Cytotoxicity and effects of cell death inhibitors**

The viability of 4T1 and MDA-MB-231 cells was assessed by using the CCK-8 assay after co-culturing them with MIL-88B, MIL-Cu_1.8_S and MIL-Cu_1.8_S-TPP/FA. Cells were harvested from the culture plates and seeded (8000 cells per well) in a 96-well plate. After incubation overnight, the media in the wells were replaced with media containing samples at various concentrations along with a negative control group. After 24 h of incubation, CCK-8 working solution was added, and the incubation was continued for an additional 2 h. Finally, the absorbance was measured at 490 nm in the microplate reader, and the percentage viability of cells in various treatments was calculated.

To investigate the cell death pathway induced by MIL-88B, MIL-Cu_1.8_S and MIL-Cu_1.8_S-TPP/FA, 4T1 cells were harvested from the culture plates and seeded (8000 cells per well) in a 96-well plate. After incubation overnight, the media in the wells were replaced with media containing Fer-1 (10 μM), Lip-1 (μM), UK5099 (μM), Rentone (3 μM), 2-thenoyltrifluoroacetone (TTFA, 100 μM), Carboxin (30 μM), Antimycin A (2.5 μM), sodium azide (NaN_3_, 5 μM), and Oligomycin (5 μM), respectively. After 1 h of pre-treatment, MIL-Cu_1.8_S-TPP/FA as well as other control groups were subsequently added to the medium with the final Cu_1.8_S concentration of 25 μg mL^−1^, where the control groups were added an equal amount of MIL-88B or Cu_1.8_S according to the iron and copper ions concentration in the MIL-Cu_1.8_S-TPP/FA. After another 24 h of incubation, CCK-8 working solution was added, and the incubation was continued for an additional 2 h. Finally, the absorbance was measured at 490 nm in the microplate reader, and the percentage viability and relative viability of cells in various treatments were calculated.

The cell death was also performed with Annexin V-FITC apoptosis detection kit (Beyotime, Shanghai, China). 4T1 cells were cultured in 6-well plate. After 24 h incubation, various samples were added into wells (50 μg mL^−1^ per well). Afterwards, 4T1 cells were collected after 24 h incubation and washed with PBS (4 °C). And cells were further centrifuged (2400 rpm, 10 min) and resuspended in 100 μL of binding buffer composed of 5 μL of Annexin V-FITC and 5 μL of PI for 10 min in the dark. Following, cells were centrifuged and resuspended in 100 μL of binding buffer to analyze by a flow cytometer.

**1.10. *In vitro* ROS level detection**

H_2_-DCFDA was used to reveal the oxidative stress after treatment of MIL-88B, MIL-Cu_1.8_S and MIL-Cu_1.8_S-TPP/FA with/without NIR light. 4T1 cells were seeded in a 12-well plate overnight. After 6 h of the introduction of respective samples at a concentration of 25 μg mL^−1^, the culture medium was replaced by 500 μL of the fresh medium without phenol red. The cells were irradiated with NIR light (1.5 W cm^−2^) for 10 min, followed by 2-h incubation. The cells were treated with DCFH_2_-DA (10 μM) and incubated at 37 °C for 30 min. The *in vitro* ROS generation was detected by a confocal laser scanning microscope (CLSM, FV1200, Olympus, Tokyo, Japan) and measured by Image J software.

**1.11. Labile iron**

4T1 cells were seeded in a 12-well plate and incubated overnight. After 1 or 3 h of the introduction of MIL-88B, MIL-Cu_1.8_S and MIL-Cu_1.8_S-TPP/FA at a concentration of 25 μg mL^−1^, the culture medium was replaced by 500 μL of the fresh medium without phenol red. The cells were treated with Calcein-AM (10 μM) and incubated at 37 °C for 30 min. The *intracellular* labile iron was reflected by a fluorescent inverted microscope and measured by Image J.

**1.12. Western blot**

4T1 cells were treated with MIL-88B, MIL-Cu_1.8_S and MIL-Cu_1.8_S-TPP/FA for 24 h and then lysed with RIPA lysis buffer containing 1% PMSF. The protein concentration was quantified using BCA. Equal amounts of cell lysates were denatured and separated by 8–15% SDS-PAGE and transferred to polyvinylidene fluoride (PVDF) membrane. After incubating in 5% skim milk powder for 2 h, the membranes were incubated overnight at 4 °C with primary antibodies. Then the PVDF membranes were further incubated with the secondary antibody at room temperature for 2 h. The immunoblots was visualized by an ECL chemiluminescence substrate kit from SageBrightness (Beijing, China). The band intensities were quantified using ImageJ software, normalized to the internal control (GAPDH).

**1.13. GSH/GSSG ratio and lipid peroxidation**

4T1 cells were seeded in a 6-well plate and incubated overnight. The cellular GSH/GSSG ratio in 4T1 cells was evaluated by measuring the levels using the GSH/GSSG assay kit after treatment with MIL-88B, MIL-Cu_1.8_S and MIL-Cu_1.8_S-TPP/FA at a concentration of 25 μg mL^−1^ with/without 808-nm light irradiation for 6 h following the manufacturer’s instructions.

The cellular lipid peroxidation in 4T1 cells was evaluated by measuring the concentration of MDA, a product of lipid peroxidation, using the lipid peroxidation MDA assay kit after treatment with MIL-88B, MIL-Cu_1.8_S and MIL-Cu_1.8_S-TPP/FA at a concentration of 25 μg mL^−1^ with/without 808-nm light irradiation at a concentration of 12.5 μg mL^−1^ for 24 h following the manufacturer’s instructions

**1.14. Data acquisition and cross-talking analysis of ferroptosis and cuproptosis regulators**

The Gene Expression Omnibus (GEO) database (GSE15852) including samples from breast cancer cases (n=86) was accessed. The expression levels of caretaker genes, constructing distribution for the expression matrix, generating hierarchical clustering and principal component analysis (PCA) were conducted to verify data integrity. All data was normalizing using the ‘limma’ R package. To investigate the relationship between ferroptotic and cuproptotic regulatory factors in breast cancer, the expression data associated with these factors were extracted and filtered. Subsequently, the correlation heatmap to elucidate the interrelationships among ferroptotic and cuproptotic regulatory genes was formed by using the ‘ComplexHeatmap’ package.

**1.15. Animal model**

Animal experiments were conducted under the guidance of protocols approved by the Research Ethics Committee of Zhejiang Sci-Tech University (20230701-73). Female nude mice with a weight of around 18 g (5-6 weeks old) were purchased from Shanghai laboratory animal Co., Ltd. and housed with free access to water and food ad libitum. To establish the 4T1 tumor model, 4T1 cells were injected subcutaneously at the right flank of mice (1×10^6^ cells per mouse, 100μL of saline). When the tumor volume reached about 80-100 mm^3^, the samples were injected intravenously through the tail vein. The tumor volume (V) was determined by measuring the length (a) and width (b), and calculated using the following:

$V=\frac{1}{2}a{\times b}^{2}$ (3)

The relative tumor growth rate was according to the formula:

$Tumor growth rate \left( \% \right)=\frac{V_{t}-V_{0}}{V_{0}}\times100\%$ (4)

where V_0_ and V_t_ are the tumor volumes at baseline and a given time point, respectively.

**1.16. Biodistribution**

4T1 tumor-bearing female mice were sacrificed at 6 h, 12 h, 18 h and 24 h after MIL-Cu_1.8_S-TPP/FA/FITC (20 mg kg^−1^, 0.1 mL) injection. The tumor tissue and major organs, including heart, liver, spleen, lung, kidney and tumor, were collected and observed by an animal bio-luminescence system (ABL-X6, Tanon, Shanghai, China).

1.15. *In Vivo* Biological Toxicity and Cancer Treatment Evaluation

*In vivo* antitumor efficacy assay was performed on 4T1 tumor bearing female mice. The mice with the tumor volume around 80-100 mm^3^ were randomly divided into 8 groups (mice in each group n = 5) and injected intravenously with PBS, MIL-88B, MIL-Cu_1.8_S and MIL-Cu_1.8_S-TPP/FA dispersion in 0.1 mL of PBS at the tail (20 mg kg^−1^) with/without an 808-nm laser (1.5 W cm^−2^) treatment for 10 min, 18 h after injection. The body weight and tumor volume for each mouse were monitored every 2 d after treatment. In addition, the major organs from sample-injected mice 14 days after treatment were collected. H&E staining of major organs and tumors were conducted.

**1.17. Statistical analyses**

All of the data were presented as mean ± standard deviation (SD). The statistical analysis was determined using one-way ANOVA analysis of variance via GraphPad Prism 9 software (*P < 0.05, **P < 0.01, ***P < 0.001, and ****P < 0.0001).


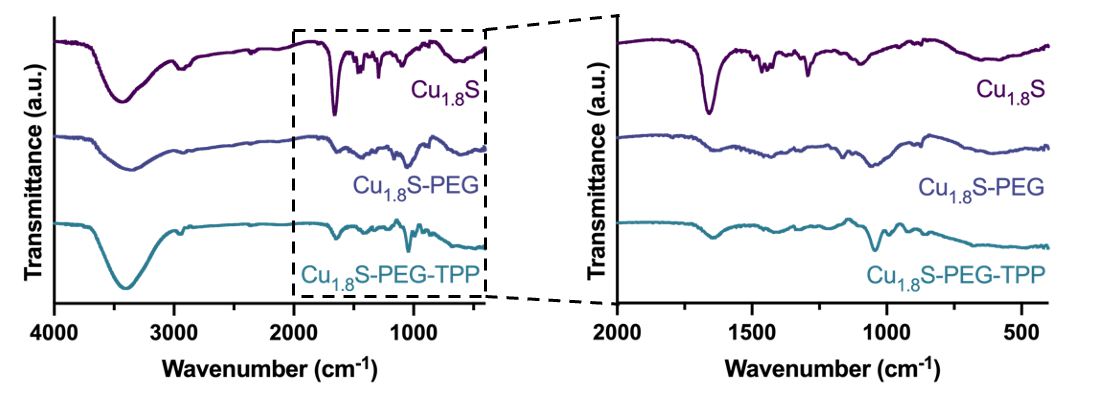


Figure S1. FTIR spectrum of Cu_1.8_S, Cu_1.8_S-PEG and Cu_1.8_S-PEG-TPP.


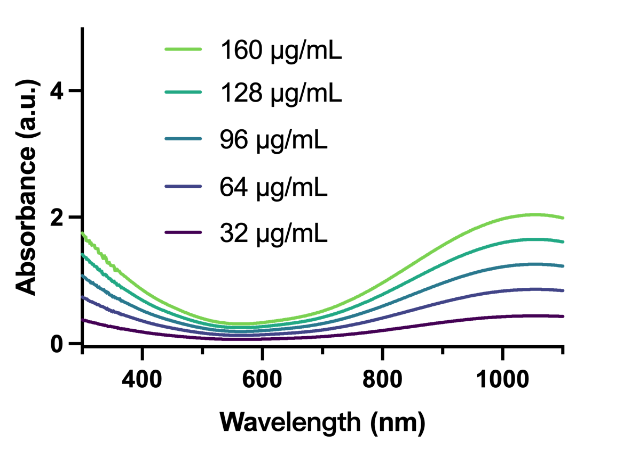


Figure S2. UV−vis spectrum of Cu_1.8_S-PEG-TPP aqueous dispersion at different concentration.


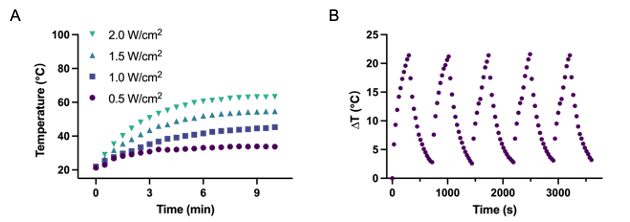


Figure S3. (A) Temperature curves of Cu_1.8_S-PEG-TPP aqueous dispersion exposed on 808-nm laser with different power. (B) Temperature curves of Cu_1.8_S-PEG-TPP aqueous dispersion exposed under 08-nm laser (1.5 W cm^−2^) with 5 temperature cycles.


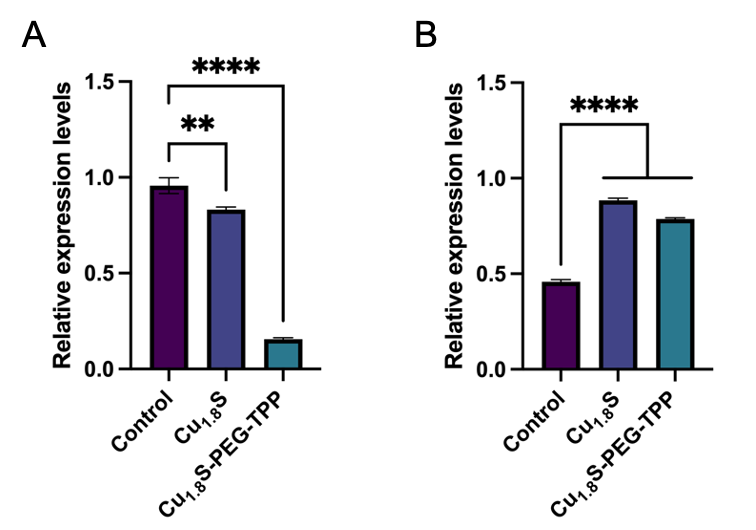


Figure S4. Quantification of (A) FDX-1 and (B) HSP70 expression levels by densitometry and normalized with GAPDH in each sample. ** *P* < 0.01, **** *P* < 0.0001.


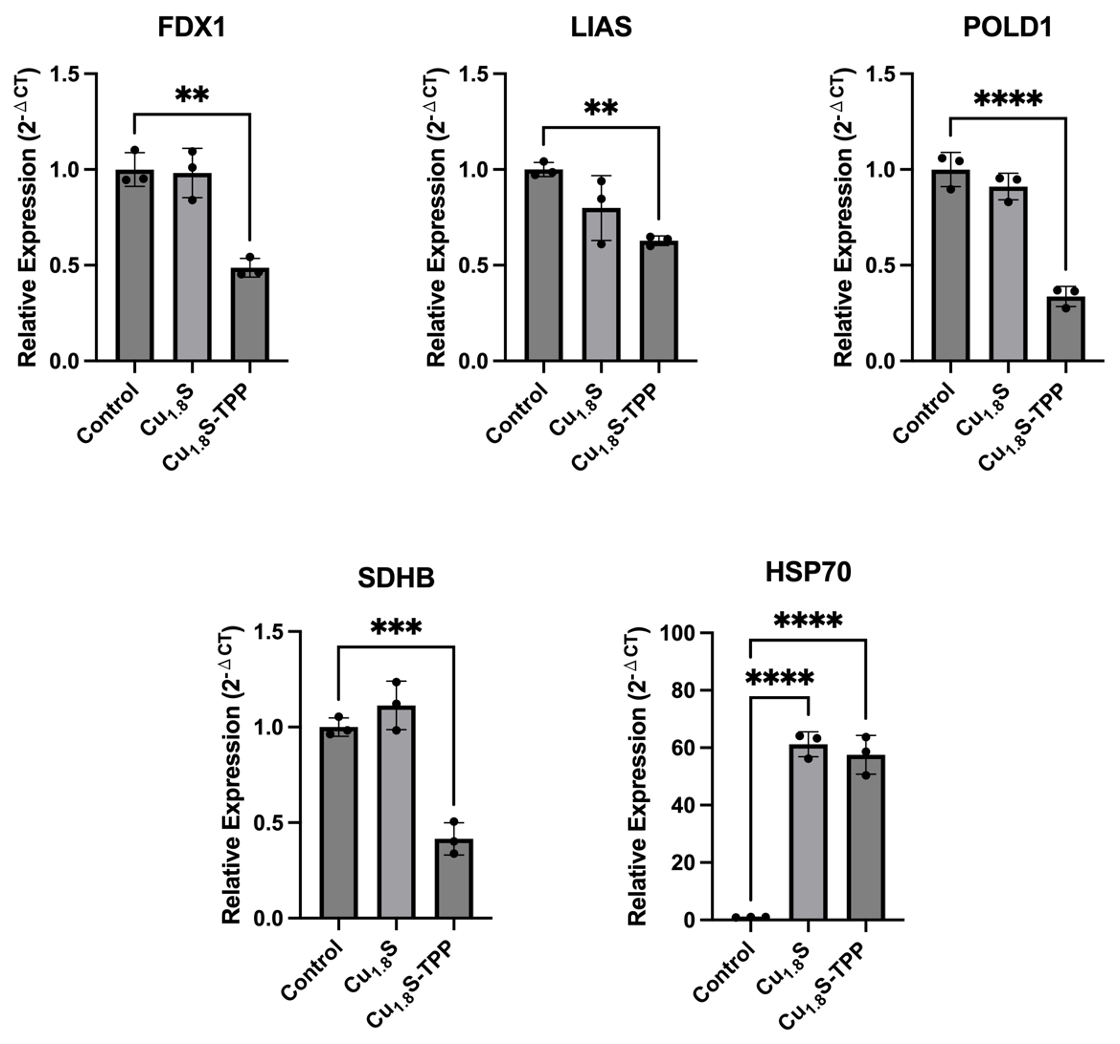


Figure S5. Relative mRNA expression of cuproptosis-related pathways, including FDX-1, LIAS, POLD1, SDHB, and HSP70, in 4T1 cells after treatments of Cu_1.8_S and Cu_1.8_S-PEG-TPP. *** *P* < 0.001, **** *P* < 0.0001.


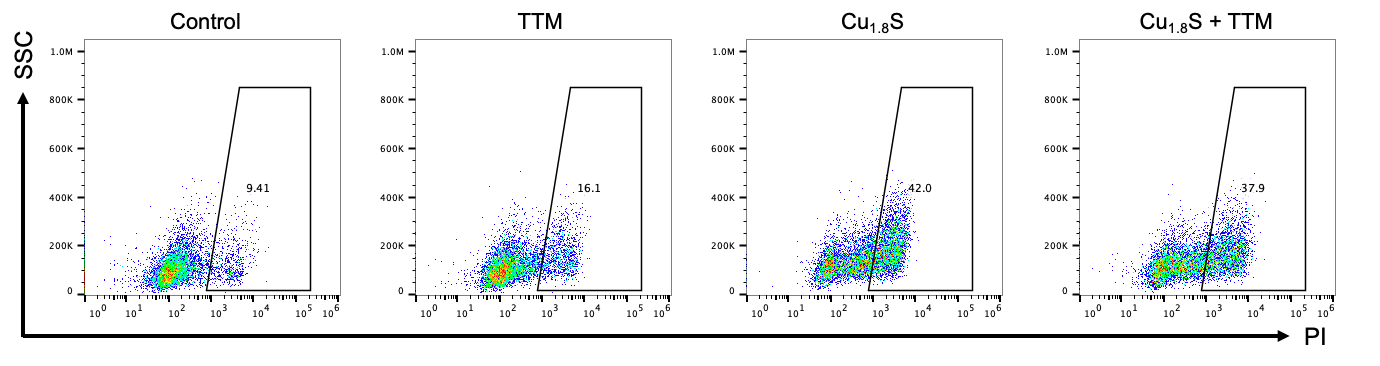


Figure S6. Cell death by detecting PI uptake of 4T1 cells after treatment of Cu_1.8_S nanodots (50 μg mL^−1^) for 24 h by using flow cytometer.


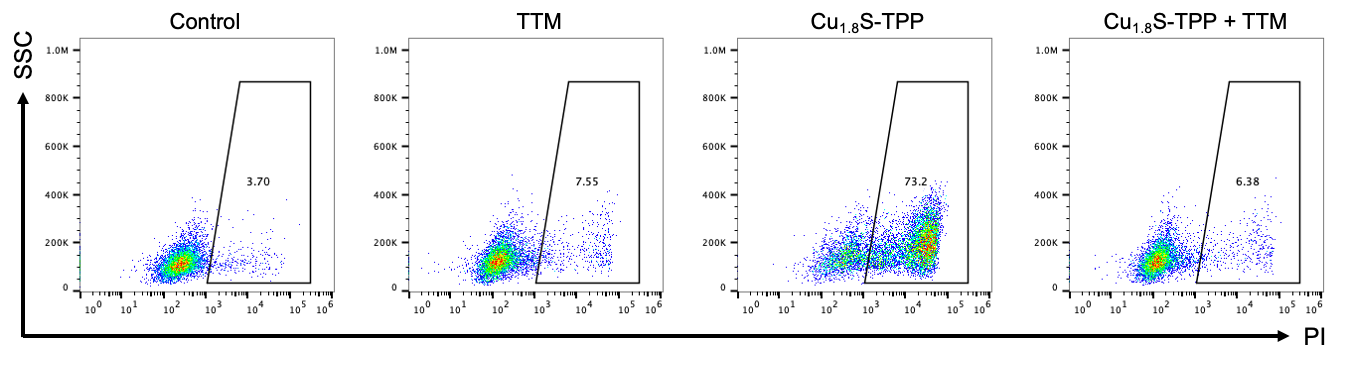


Figure S7. Cell death by detecting PI uptake of 4T1 cells after treatment of Cu_1.8_S-TPP (50 μg mL^−1^) for 24 h by using flow cytometer.


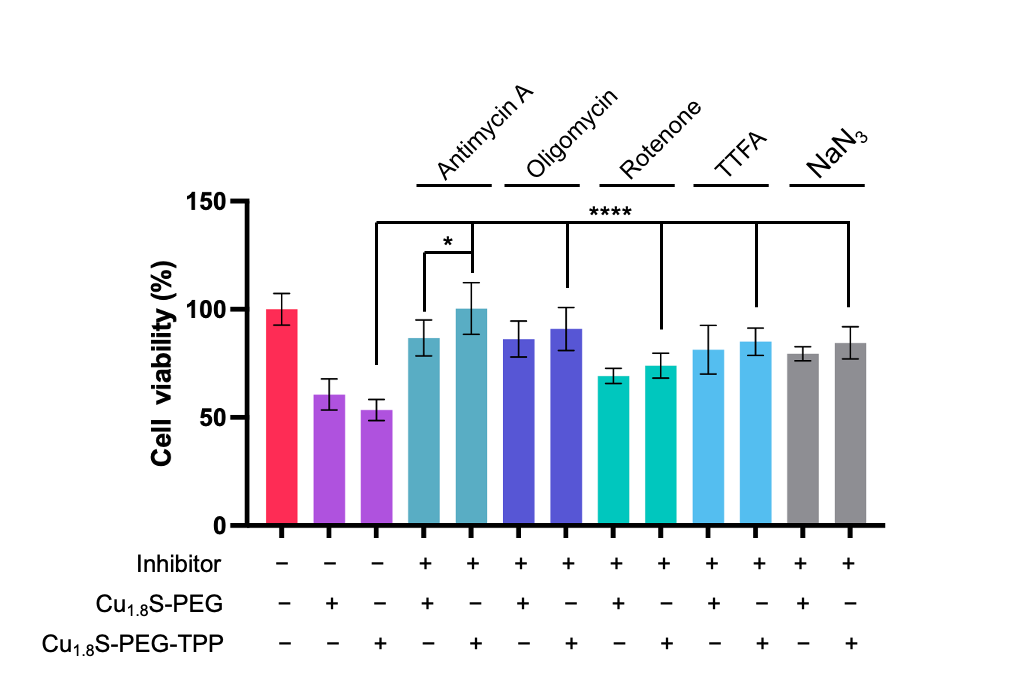


Figure S8. Cell viability of MB-MDA-231 cells pretreated with various inhibitors, including antimycin A, Oligomycin, rentone, TTFA, and NaN_3_, and then treated with Cu_1.8_S and Cu_1.8_S-PEG-TPP. * *P* < 0.05 and **** *P* < 0.0001.


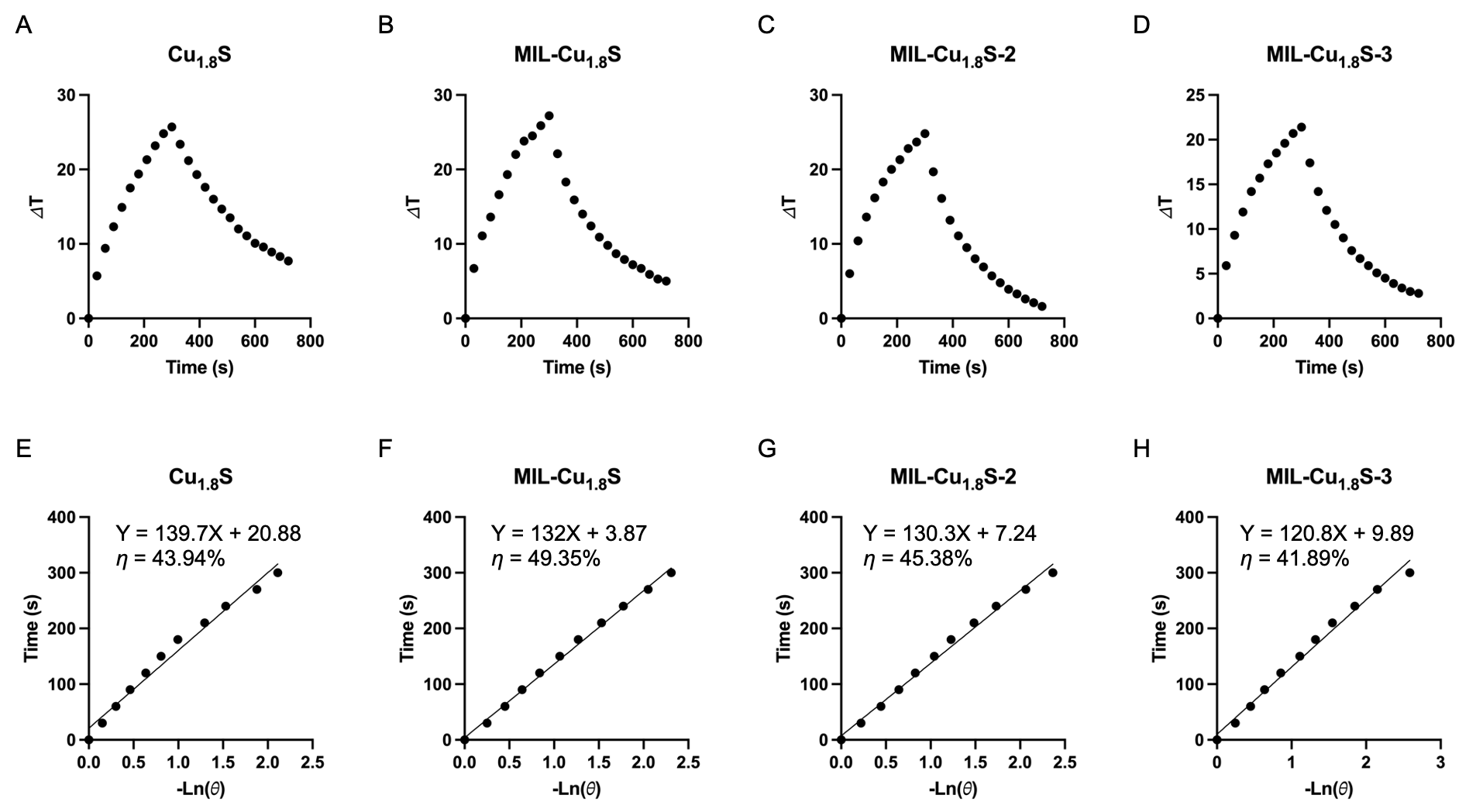


Figure S9. (A-D) The temperature change (ΔT) response of Cu_1.8_S, MIL-Cu_1.8_S, MIL-Cu_1.8_S-2, and MIL-Cu_1.8_S-3 with 808 nm laser irradiation on and off in period of 660 s (Concentration of Cu_1.8_S in all samples is 100 μg mL^−1^, and the w/w proportions of Cu_1.8_S in the whole MIL-Cu_1.8_S, MIL-Cu_1.8_S-2 and MIL-Cu_1.8_S-3 was 24.79%, 28.2% and 32.0%, respectively). (E-F) The respective linear time data versus-ln (*θ*) obtained from the cooling period of NIR laser off.


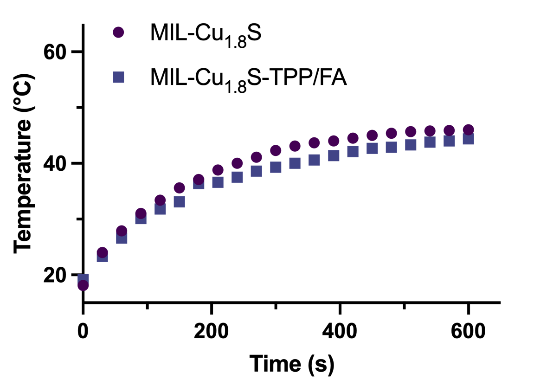


Figure S10. Temperature curves of MIL-Cu_1.8_S and MIL-Cu_1.8_S-TPP/FA aqueous dispersion exposed on 808-nm laser (1.5 W cm^−2^).


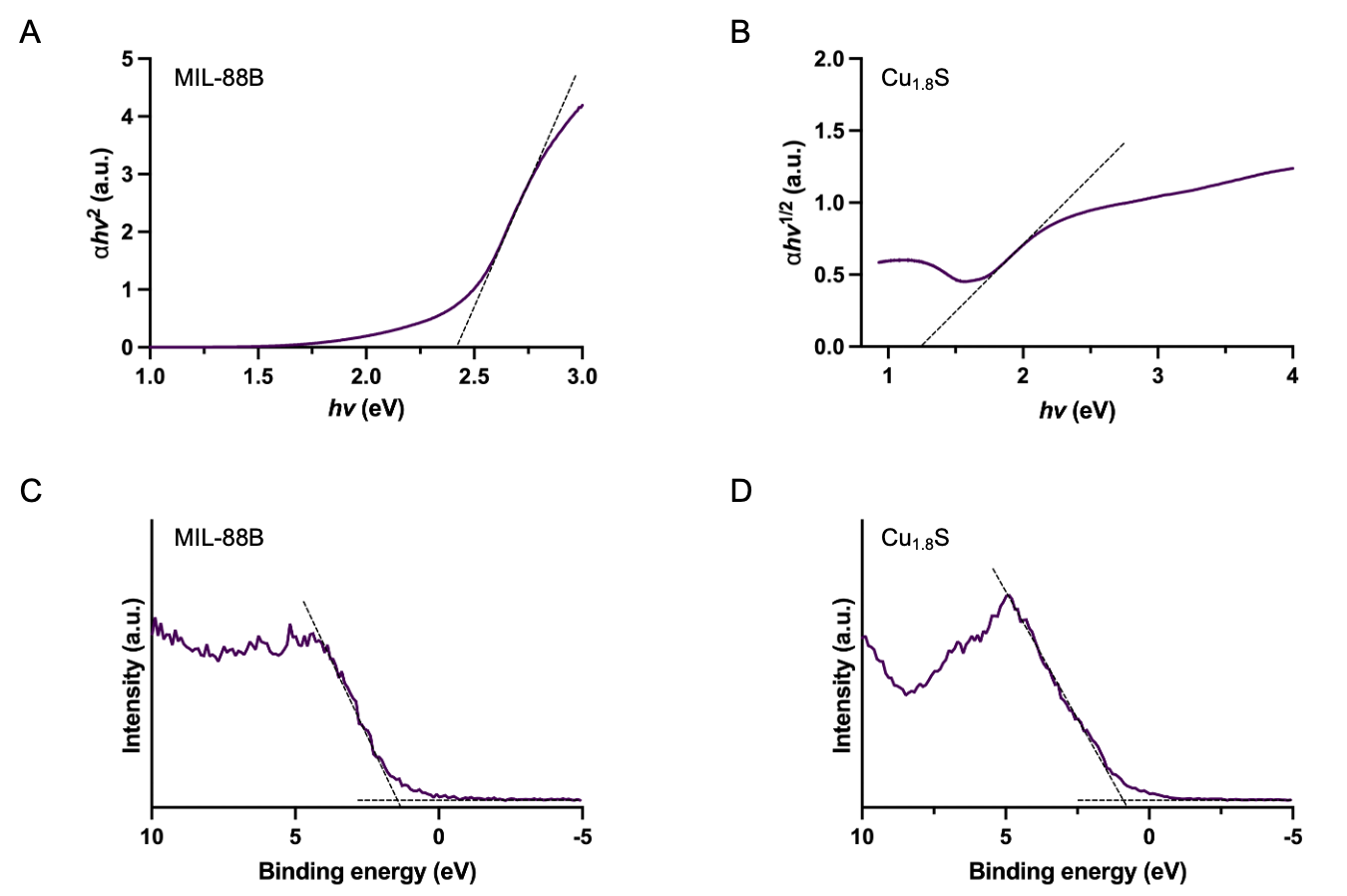


Figure S11. (A) The (A*hν*)^2^ versus *hν* curve of MIL-88B. (B) The (A*hν*)^1/2^ versus *hν* curve of Cu_1.8_S. Valence-band XPS spectrum of (C) MIL-88B and (D) Cu_1.8_S.


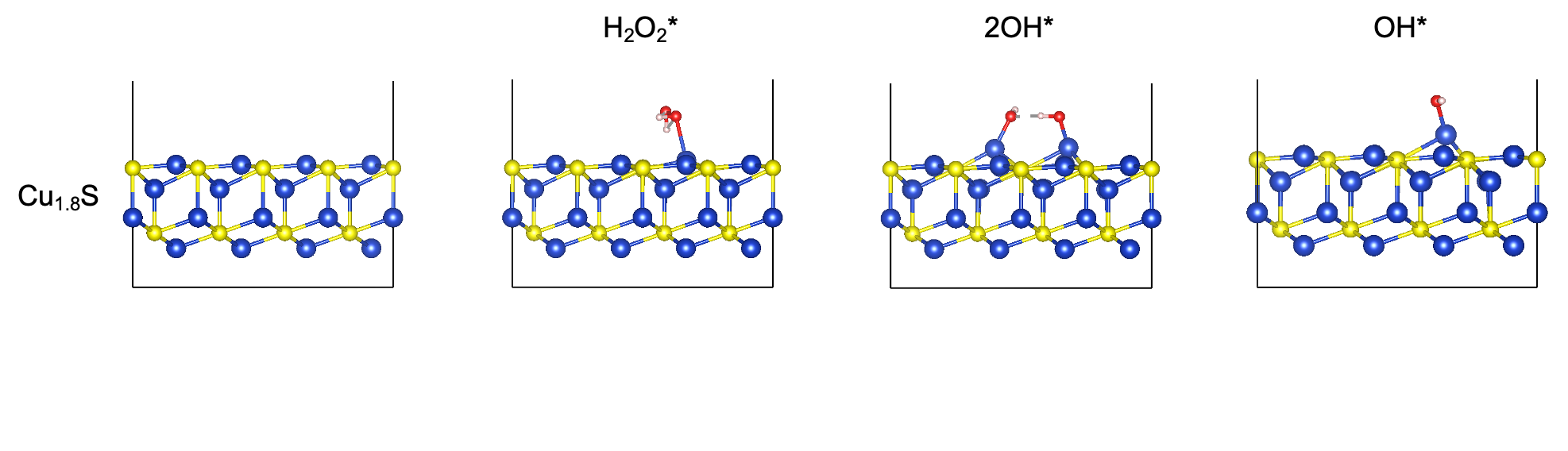


Figure S12. Cu_1.8_S models in the prior H_2_O_2_-dissociation process.


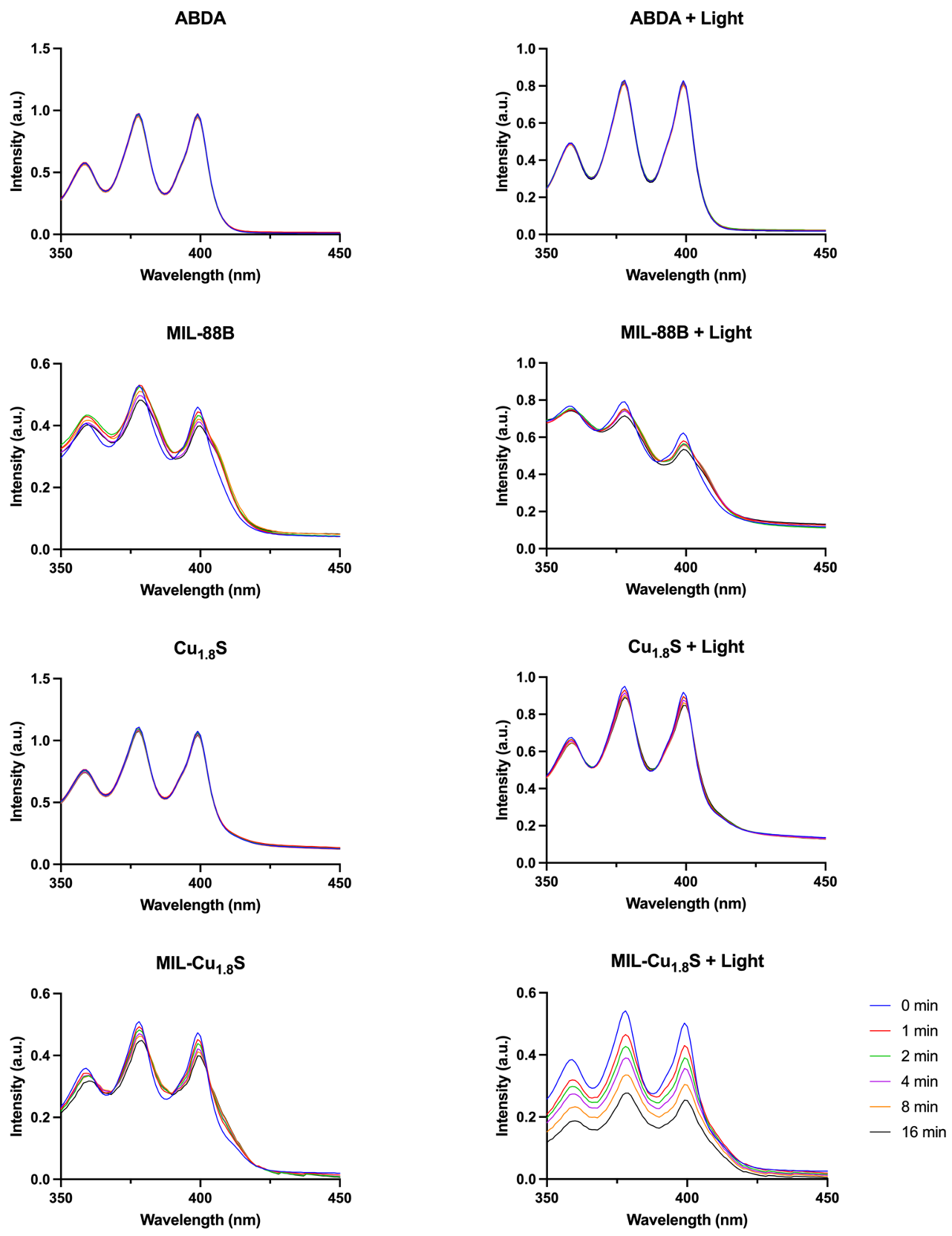


Figure S13. ^1^O_2_ generation of MIL-88B, Cu_1.8_S and MIL-Cu_1.8_S with or without 808-nm laser irradiation.


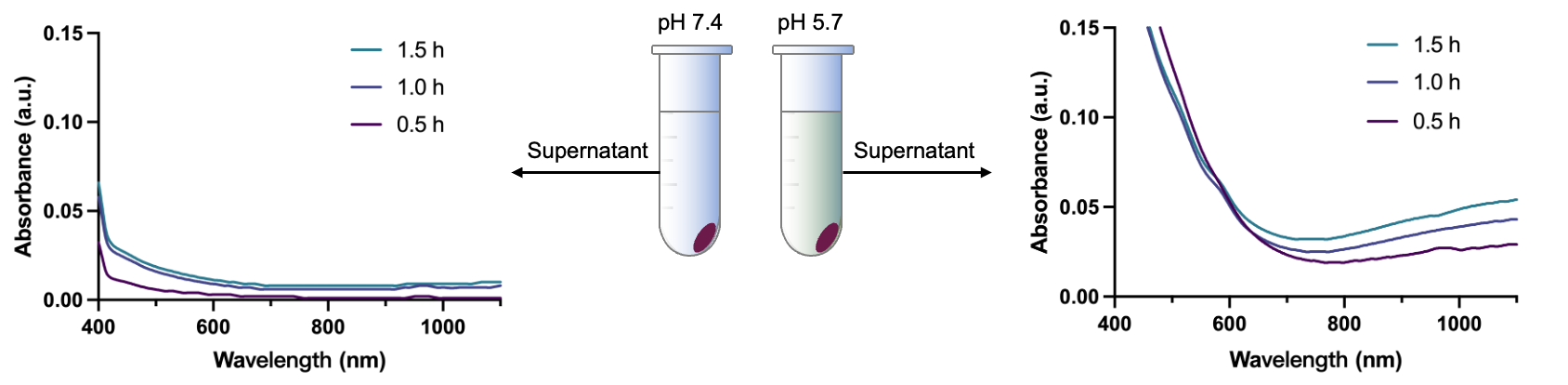


Figure S14. The dissociation of Cu_1.8_S nanodots from MIL-Cu_1.8_S in different environment.


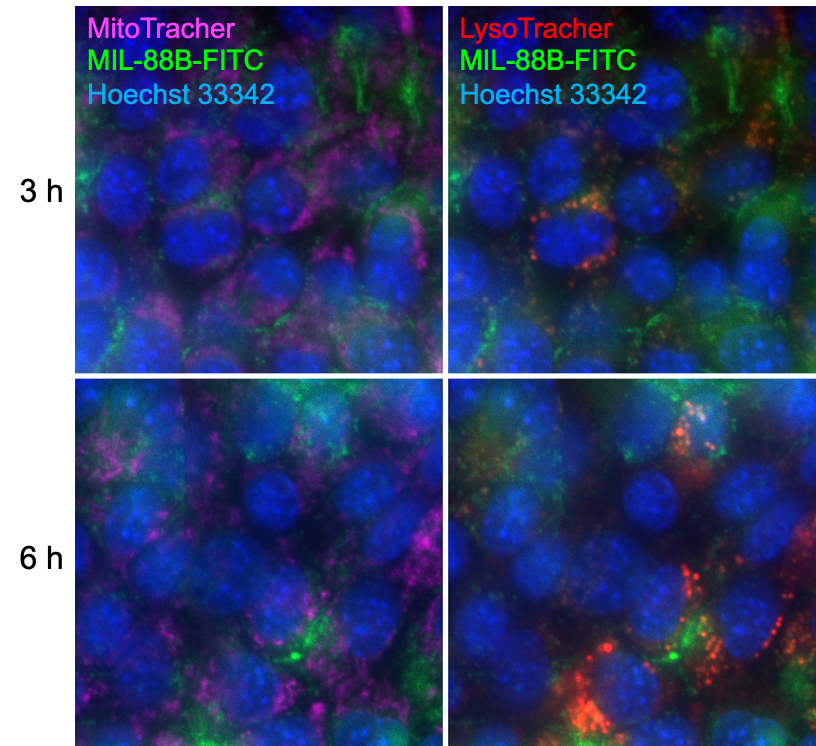


Figure S15. CLSM images of 4T1 cells after 3-h or 6-h co-incubation with FITC@MIL-Cu_1.8_S-TPP/FA.


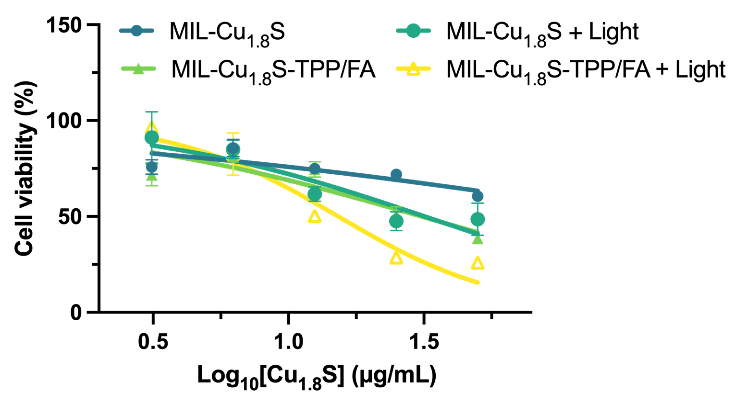


Figure S16. Cell viability of MIL-Cu_1.8_S and MIL-Cu_1.8_S-TPP/FA in MB-MDA-231 cell line.


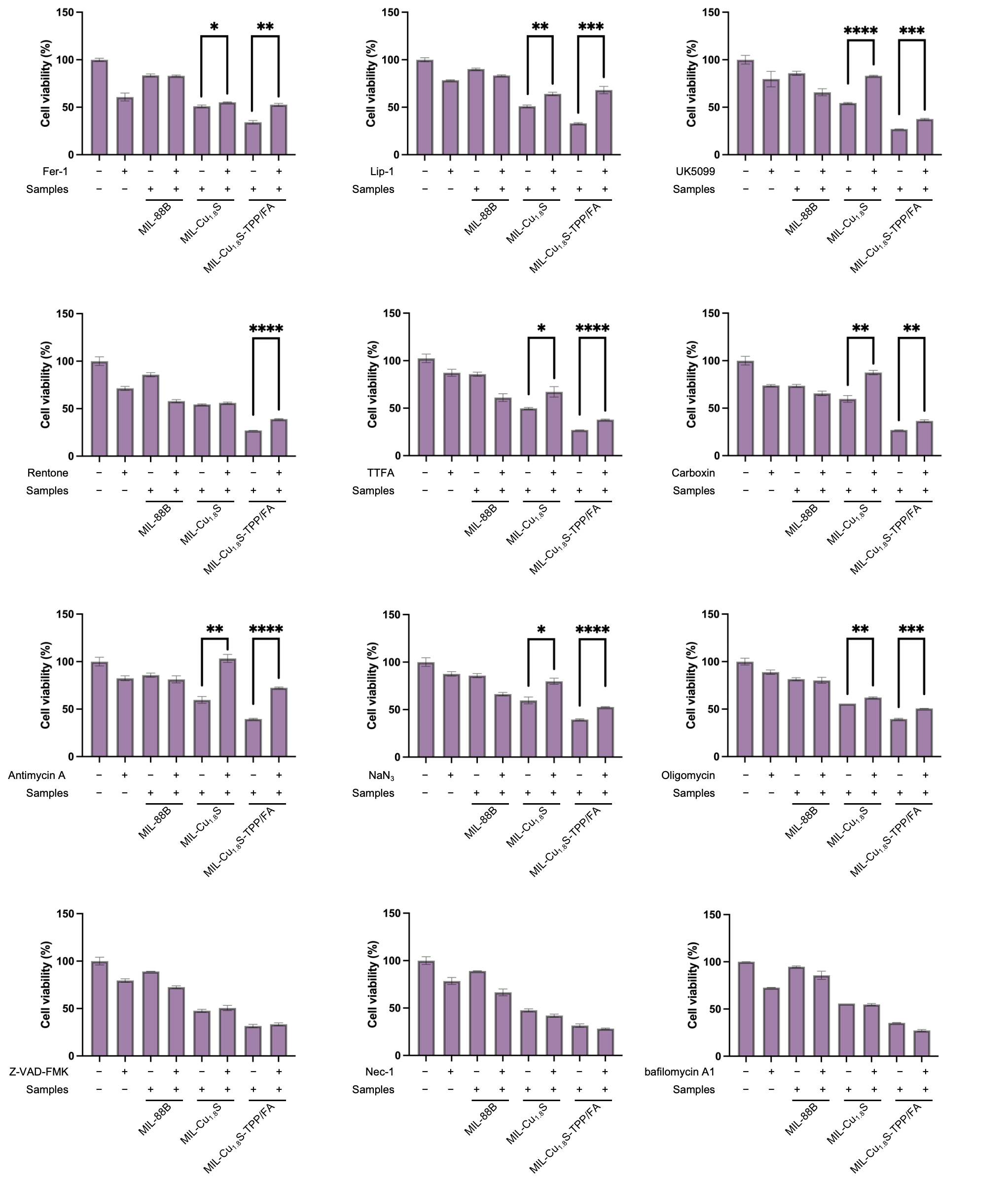


Figure S17. Cell viability of 4T1 cells pretreated with various inhibitors, including Fer-1, Lip-1, UK5099, rentone, TTFA, carboxin, antimycin A, NaN_3_, and oligomycin, Z-VAD-FMK, Nec-1 and bafilomycin A1, and then treated with MIL-88B, MIL-Cu_1.8_S and MIL-Cu_1.8_S-TPP/FA.


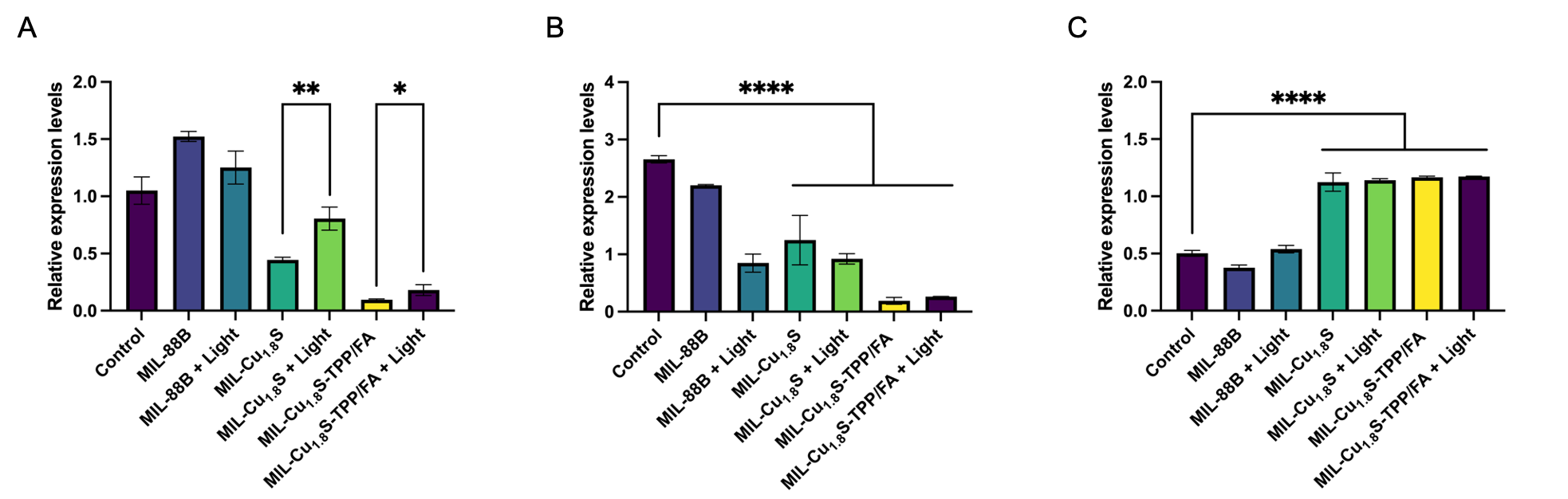


Figure S18. Quantification of (A) GPX-4 (B) FDX-1 and (C) HSP70 expression levels by densitometry and normalized with GAPDH in each sample. * < 0.05, ** *P* < 0.01, **** *P* < 0.0001.


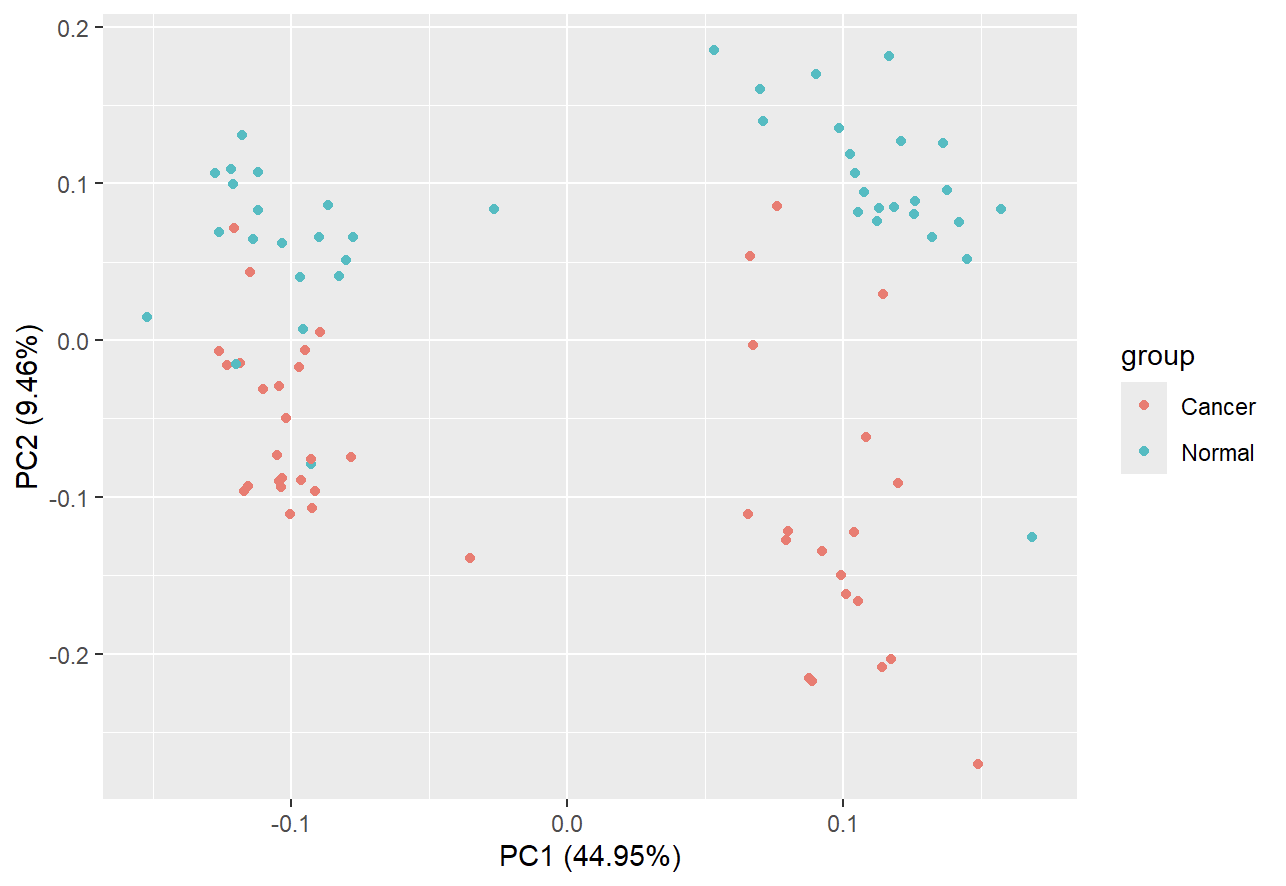


Figure S19. PCA revealed difference between cancer and normal cases.


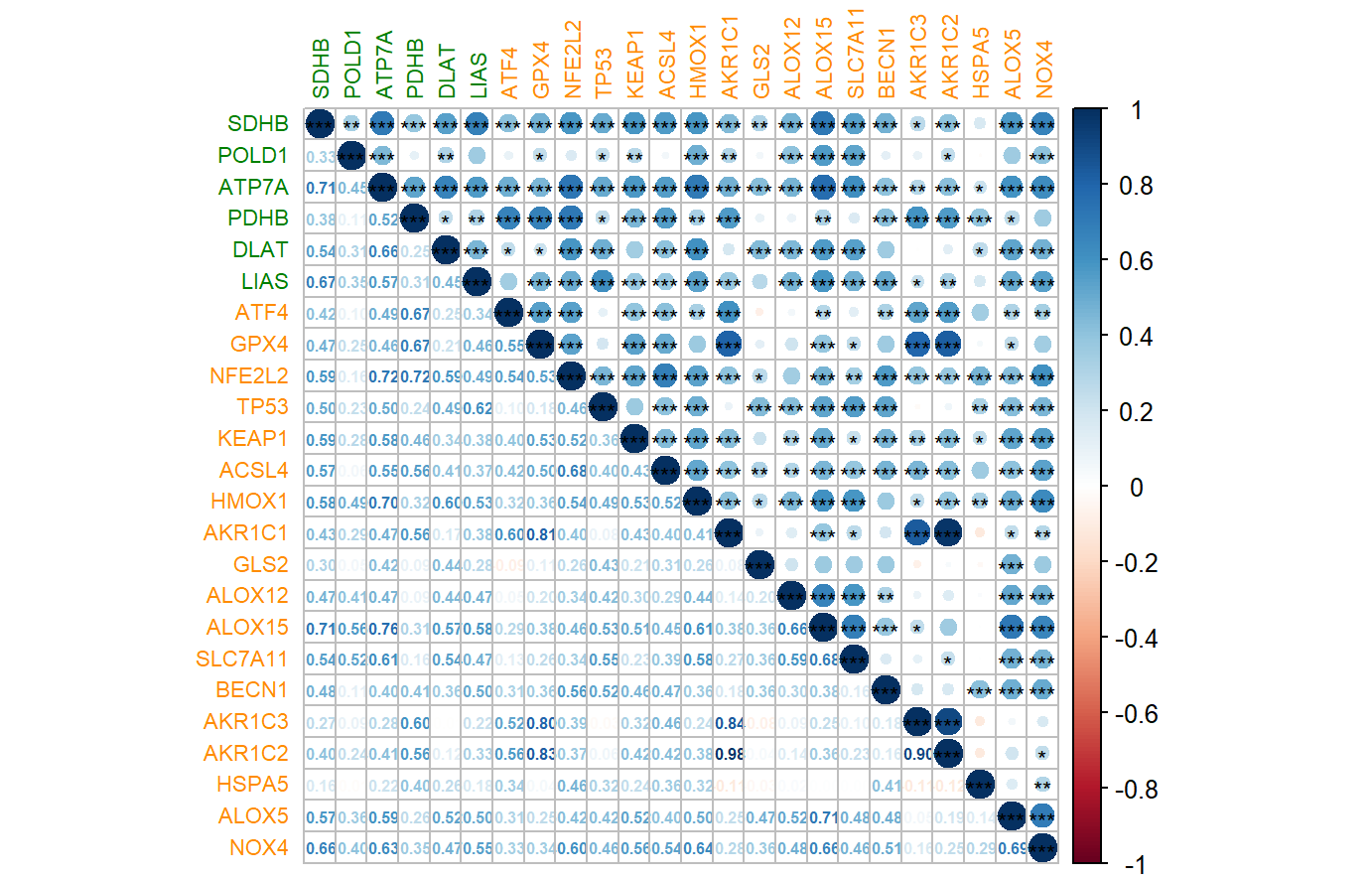


Figure S20. Co-occurrence of genetic alterations in the cuproptosis and ferroptosis regulators for breast cancer.


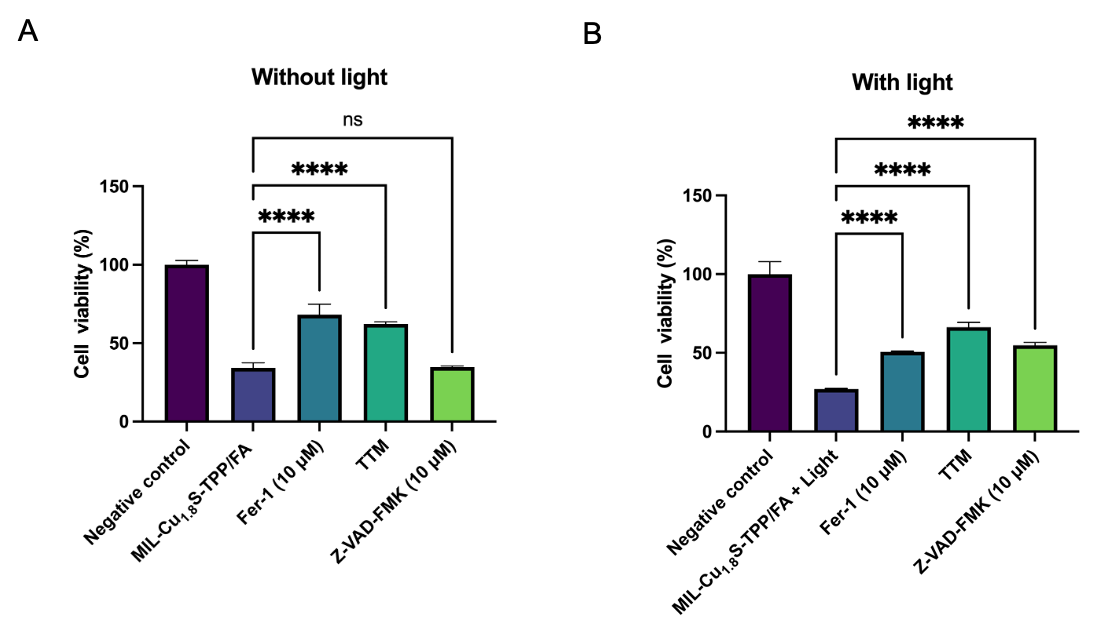


Figure S21. Cell viability of 4T1 cells treated with inhibitors of ferroptosis, cuproptosis and apoptosis, and then treated with MIL-Cu_1.8_S-TPP/FA with/without an 808-nm light irradiation.


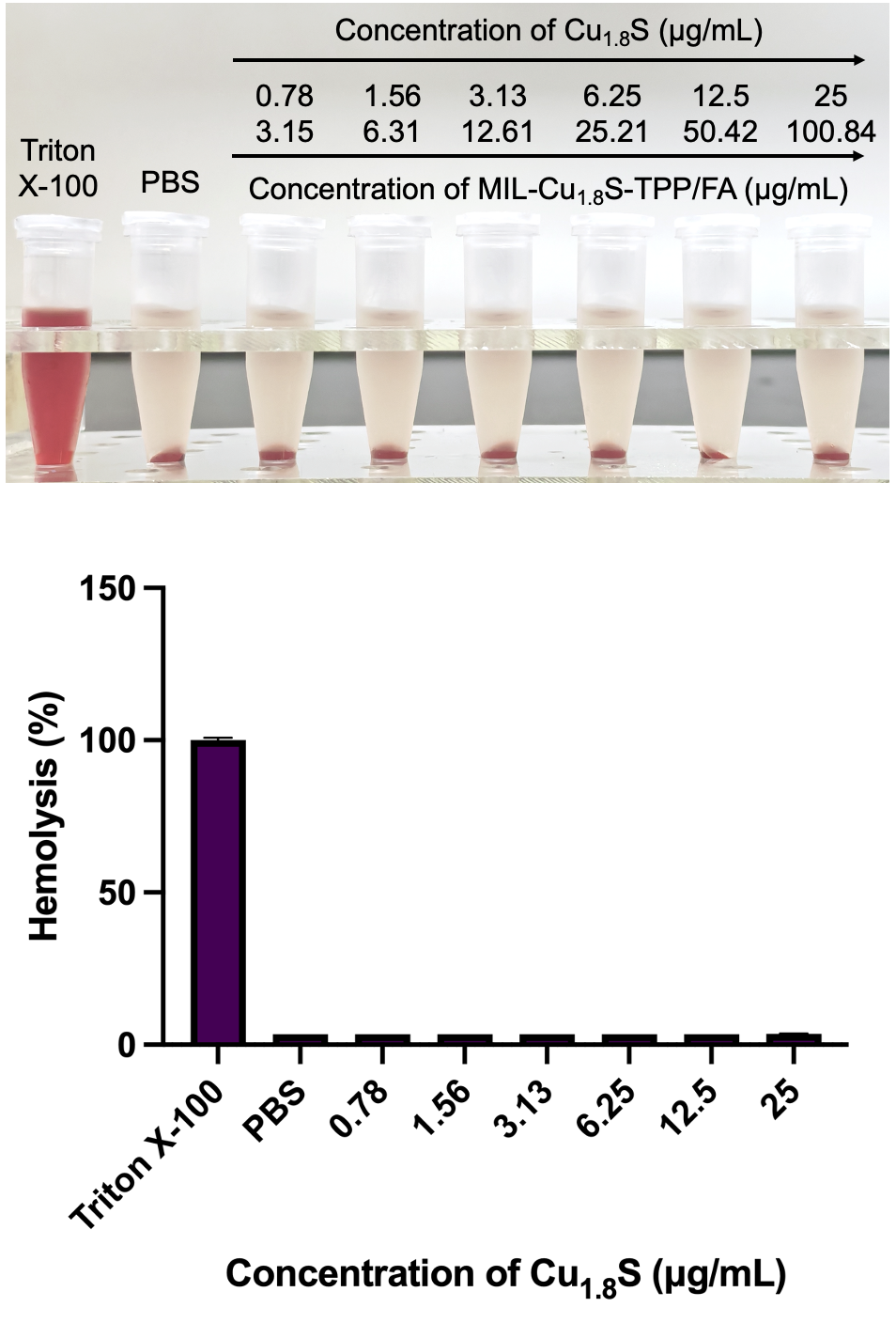


Figure S22. Photograph of hemolysis test and hemolysis rate.


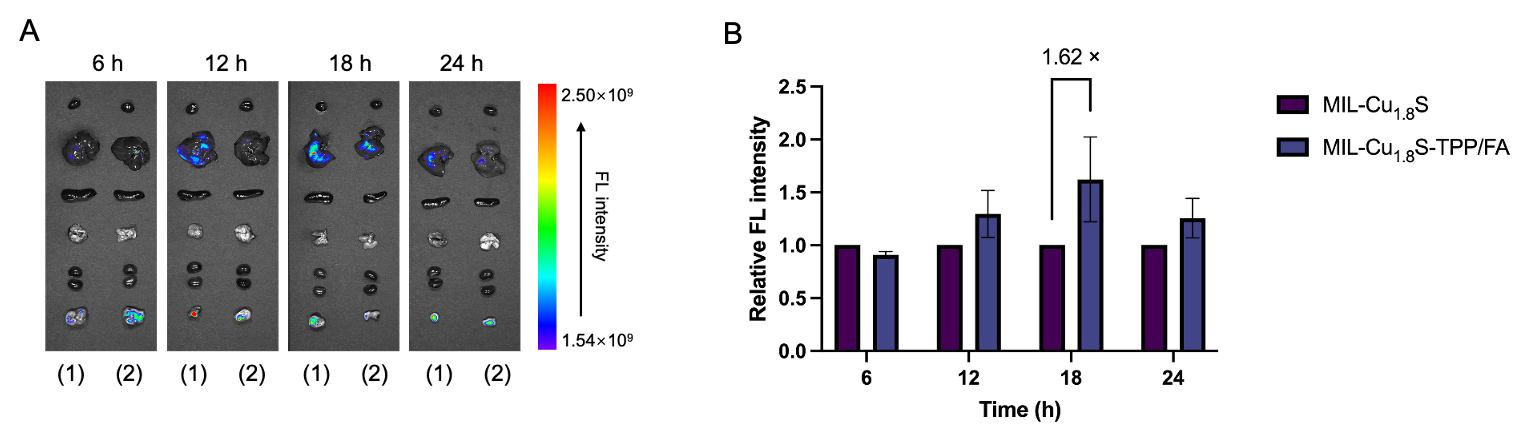


Figure S23. (A) Fluorescence images of major organs and tumors of mice treated with (1) MIL-Cu_1.8_S or (2) MIL-Cu_1.8_S-TPP/FA at different time points after tail-vein injection. (B) Relative fluorescence intensity of tumor at different time points after tail-vein injection of samples.


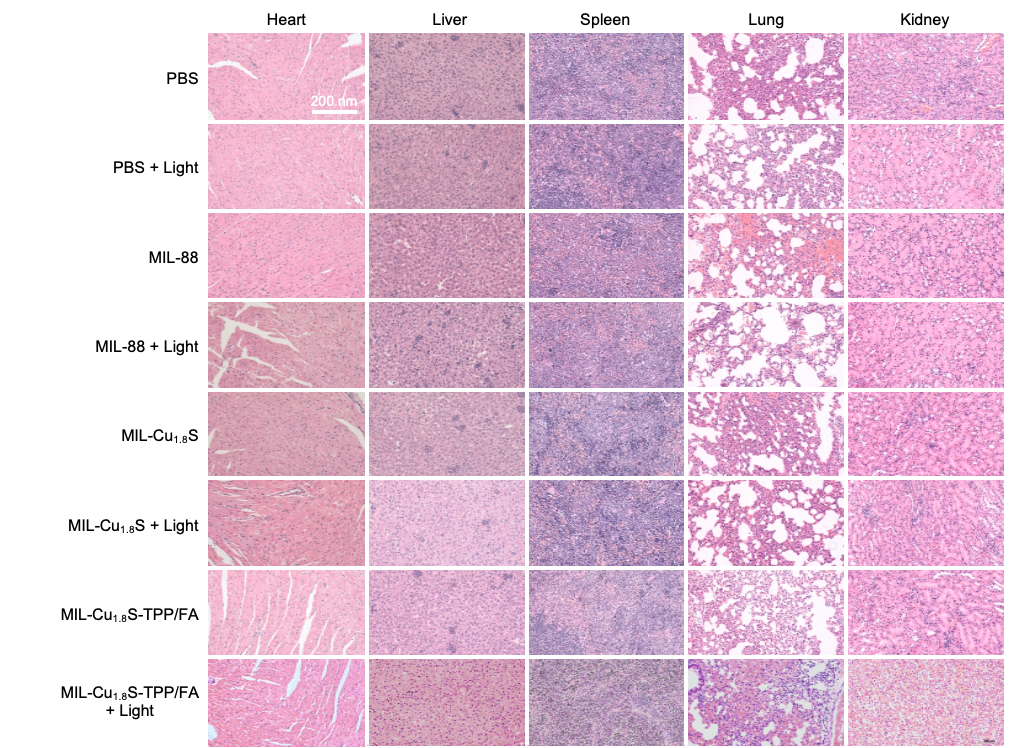


Figure S24. H&E staining images of major organs and tumors after 14 d treatment with PBS, MIL-88B, MIL-Cu_1.8_S and MIL-Cu_1.8_S-TPP/FA with/without NIR light irradiation.
